# Imaging strategies for patients with multiple and/or severe injuries in the resuscitation room: a systematic review and clinical practice guideline update

**DOI:** 10.1007/s00068-025-02840-8

**Published:** 2025-04-02

**Authors:** Stefan Huber-Wagner, Rainer Braunschweig, Daniela Kildal, Dan Bieler, Barbara Prediger, Miriam Hertwig, Charlotte Kugler, Stefan Reske, Thomas Wurmb, Gerhard Achatz, Benedikt Friemert, Carsten Schoeneberg

**Affiliations:** 1 Department of Trauma Surgery, Schwäbisch Hall Diakonie Hospital, Schwäbisch Hall, Germany; 2 Institut of Radiology, University Erlangen, Germany, Member of the Board of the Working Group On Musculoskeletal Imaging (AG MSK) of the German Radiological Society, Berlin, Germany; 3 Department of Radiology, Upper Valais Hospital Centre (Brig), Brig, Switzerland; 4 Department of Orthopaedics and Trauma Surgery, Medical Faculty University Hospital Duesseldorf, Heinrich-Heine-University, Moorenstrasse 5, 40225 Duesseldorf, Germany; 5 Department of Trauma Surgery and Orthopaedics, Reconstructive Surgery, Hand Surgery and Burn Medicine, German Armed Forces Central Hospital Koblenz, Koblenz, Germany; 6 Institute for Research in Operative Medicine (IFOM), Witten/Herdecke University, Cologne, Germany; 7 Department of Radiology, Heinrich Braun Hospital, Zwickau, Germany; 8 Department of Anaesthesiology, Intensive Care, Emergency Medicine and Pain Therapy, Würzburg University Hospital, Würzburg, Germany; 9 Department of Trauma Surgery and Orthopaedics, Reconstructive and Septic Surgery, Sports Traumatology, Bundeswehr Hospital of Ulm, Ulm, Germany; 10 Bundeswehr Hospital of Ulm, Ulm, Germany; 11 Department of Orthopaedics and Trauma Surgery, Alfried Krupp Hospital, Essen, Germany

**Keywords:** Imaging strategy, Computed tomography, Polytrauma guideline, Severely injured, Diagnostic imaging, Whole-body CT

## Abstract

**Purpose:**

Our aim was to develop new evidence-based and consensus-based recommendations for imaging strategies in patients with multiple and/or severe injuries in the resuscitation room. This guideline topic is part of the 2022 update of the German Guideline on the Treatment of Patients with Multiple and/or Severe Injuries.

**Methods:**

MEDLINE and Embase were systematically searched to August 2021. Inclusion criteria: patients with multiple and/or severe injuries in the resuscitation room, randomised controlled trials, prospective cohort studies, cross-sectional studies, and comparative registry studies; comparison of interventions for imaging strategies; patient-relevant clinical outcomes such as diagnostic test accuracy and mortality. Further literature reports were obtained from clinical experts. We considered patient-relevant clinical outcomes such as diagnostic test accuracy and mortality. Risk of bias was assessed using NICE 2012 checklists. The evidence was synthesised narratively, and expert consensus was used to develop recommendations and determine their strength.

**Results:**

Twenty-one studies with a total of 55,227 patients were identified. There were studies with low risk (n = 2), high risk (n = 5) and unclear risk of bias (n = 14). Relevant topics were sonographic imaging of the chest and abdomen (n = 8 studies), X-ray of the chest (n = 1), indications for whole-body computed tomography (n = 6), CT scanner location (n = 1), whole-body computed tomography in haemodynamically unstable patients (n = 3), and prehospital sonography (n = 2). There were studies with low risk (n = 2), high risk (n = 5) and unclear risk of bias (n = 14). One new recommendation was developed, six were modified. All achieved strong consensus.

**Conclusion:**

While extended focused assessment with sonography for trauma should be performed for diagnostic purposes after blunt and/or penetrating thoracic and/or abdominal trauma as part of the primary survey in the resuscitation room, whole-body computed tomography (WBCT) gains highest importance as part of the diagnostic procedures for severely injured patients. WBCT with a trauma-specific protocol must be performed in a timely manner if the patient does not require an immediate intervention. Magnetic resonance imaging can be indicated as a further primary diagnostic tool for specific conditions. Two studies were judged to be of low risk of bias in all domains. The risk of selection bias was high in two studies and unclear in seven studies.

**Supplementary Information:**

The online version contains supplementary material available at 10.1007/s00068-025-02840-8.

## Introduction

For more than 15 years, whole-body computed tomography (WBCT) has played an established role in the initial management of severely injured patients in the resuscitation room. Besides WBCT, the use of ultrasound plays a crucial role, particularly in detecting immediately life-threatening injuries during the primary survey or in the pre-hospitaL phase. Apart from other factors, rapid and accurate diagnostic imaging is an important prerequisite for the appropriate management of severely injured patients in the resuscitation room setting.

Technical prerequisites are high temporal resolution (subsecond scanning), relevant spatial resolution (isotropic voxels), effective contrast-medium management (split-bolus protocols), state-of-the-art dose reduction techniques (tube current and tube voltage modulation, iterative reconstruction), single-plane 3D reformatting, and a WBCT z-axis volume of more than 1.5 m in 90 s [1–4]. Once these requirements have been met, WBCT is technically possible [5, 6] and clinically useful [7–10].

In 1977, Löw et al. [11] were the first to report on the use of WBCT in the management of severely injured patients. Scherer et al. [12], Leidner and Beckman [7], Ptak et al. [9], Klöppel et al. [13], and Rieger et al. [10] too investigated potential uses of WBCT. Further publications emphasised the effectiveness of diagnostic WBCT and the role of WBCT in the management of polytrauma patients in the resuscitation room setting [8, 14–20].

A large number of trauma centres now use WBCT as a routine diagnostic tool in the management of polytrauma patients in the resuscitation room [16, 17]. According to the 2021 Annual Report of the TraumaRegister^®^ of the German Trauma Society, 77% of all hospitals participating in the trauma registry use WBCT as a diagnostic imaging tool [21]. On average, WBCT is performed within the first 25 min of admission to the resuscitation room [21]. The diagnostic value of CT imaging is undisputed; however, the necessary time consumption must be considered, especially in hemodynamically unstable patients and the optimal time frame in the trauma algorithm in the initial treatment phase has to be detected.

The core purpose of effective diagnostic imaging tests in the resuscitation room is to detect all pathological findings in a sensitive and specific manner and to distinguish between acute injuries, pre-existing conditions, and conditions that are unrelated to trauma [22–28].

The objective of this review is to analyse the evidence for diagnostic imaging studies in severely injured patients in the initial treatment phase on the basis of the existing literature. Evidence-based key recommendations on imaging modalities such as ultrasound, conventional radiography of the chest and pelvis, computed tomography, and magnetic resonance imaging are provided. They have been developed on the basis of a systematic literature review, which is described in the Methods section.

These key recommendations apply to severely injured patients aged 15 years or older. Recommendations on diagnostic imaging modalities for younger patients are provided in the S2k Guideline on the Management of Paediatric Polytrauma Patients of the Committee on Paediatric Traumatology of the German Trauma Society [29].

## Methods

This guideline topic is part of the 2022 update of the German Guideline on the Treatment of Patients with Multiple and/or Severe Injuries [30]. The guideline update is reported according to the RIGHT tool [31], the systematic review part according to the Preferred Reporting Items for Systematic Reviews and Meta-Analyses (PRISMA) 2020 reporting guideline [32]. The development and updating of recommendations followed the standard methodology set out in the guideline development handbook issued by the German Association of the Scientific Medical Societies (AWMF) [33]. All methods were defined a priori, following the methods report of the previous guideline version from July 2016 [34] with minor modifications, as detailed below. The publication as a systematic review has the advantage that parts of the method report, the guideline chapter and the evidence tables are directly related to each other so that the reader, unlike the guideline, gets a clear overview of all these aspects in one work. This approach was chosen, among other things, to increase the implementation of the guideline content overall [30].

### PICO questions and eligibility criteria

Population, intervention, comparison, and outcome (PICO) questions were retained from the previous guideline version. In addition, the participating professional societies involved in guideline development were asked to submit new PICO questions. The overarching PICO question for this topic area was:

*In adult patients (*≥*14 years) with known or suspected polytrauma and/or severe injuries, do specific imaging strategies in the resuscitation room improve patient-relevant outcomes or examination results compared to any other imaging strategy?*

The full set of pre-defined PICO questions is listed in Table S1 (Online Resource 1). The study selection criteria in the PICO format are shown in Table 1.

**Table 1:** Pre-defined selection criteria

Population:	Adult patients (≥ 14 years) with polytrauma and/or severe injuries^a^
Intervention/comparison:	Imaging in the resuscitation room (incl. organisational aspects, structural arrangements, etc.)
Outcomes:	Any patient-relevant outcome, such as diagnostic test accuracy, mortality, length of stay, function
Study type:	Comparative, prospective studies (randomised controlled trials, cohort studies) Comparative registry^b^ data (incl. case–control studies) Cross-sectional studies (only diagnostic studies) Systematic reviews based on the above primary study types
Language:	English or German
Other inclusion criteria:	Full text of study published and accessible Study matches pre-defined PICO question
Exclusion criteria:	Multiple publications of the same study without additional information

^a^Defined by an Injury Severity Score (ISS) > 15, Glasgow Coma Scale (GCS) < 9, or comparable values on other scales, or, in the prehospital setting, clinical suspicion of polytrauma/severe injury with a need for life-saving interventions

^b^Using the Agency for Healthcare Research and Quality (AHRQ) definition of registries [35]

### Literature search

An information specialist systematically searched for literature in MEDLINE (Ovid) and Embase (Elsevier). The search strategy described in the 2016 Guideline was used with modifications. It contained index (MeSH/Emtree) and free text terms for the population and intervention. The searches were completed on 31 August 2021. The start date for update searches was 1 January 2014. Table S2 (Online Resource 1) provides details for all searches. Clinical experts were asked to submit additional relevant references.

### Study selection

Study selection was performed by two reviewers in a two-step process using the predefined eligibility criteria: (1) title/abstract screening of all references retrieved from database searches using Rayyan software [36] and (2) full-text screening of all articles deemed potentially relevant by at least one reviewer at the title/abstract level in Endnote (Endnote, Version: 20 [Software]. Clarivate, Boston, Massachusetts, USA. https://endnote.com/). Disagreements were resolved through consensus or by consulting a third reviewer. The reasons for full-text exclusion were recorded (Table S3, Online Resource 1).

### Assessment of risk of bias and level of evidence

Two reviewers sequentially assessed the risk of bias of included studies at study level using the relevant checklists from the NICE guidelines manual 2012 [37] and assigned each study an initial level of evidence (LoE) using the Oxford Centre for Evidence-based Medicine Levels of Evidence (2009) [38]. For studies with baseline imbalance and unadjusted analyses, post-hoc secondary analyses, indirectness of the study population, or low power and imprecision of the effect estimate, the level of evidence was downgraded and marked with an arrow (↓). Any disagreements were resolved through consensus or by consulting a third reviewer.

### Data extraction and data items

Data were extracted into a standardised data table by one reviewer and checked by another. A predefined data set was collected for each study, consisting of study characteristics (study type, aims, setting), patient selection criteria and baseline characteristics (age, gender, injury scores, other relevant variables), intervention and control group treatments (including important co-interventions, index and reference tests for diagnostic studies), patient flow (number of patients included and analysed), matching/adjusting variables, and data on outcomes for any time point reported.

### Outcome measures

Outcomes were extracted as reported in the study publications. For prospective cohort studies and registry data, preference was given to data obtained after propensity-score matching or statistical adjustment for risk-modulating variables over unadjusted data.

### Synthesis of studies

Studies were grouped by interventions. An interdisciplinary expert group used their clinical experience to synthesise studies narratively by balancing beneficial and adverse effects extracted from the available evidence. Priority was given to diagnostic test accuracy, reducing mortality, immediate complications, and long-term adverse effects. Clinical heterogeneity was explored by comparing inclusion criteria and patient characteristics at baseline as well as clinical differences in the interventions and co-interventions.

### Development and updating of recommendations

For each PICO question, the following updating options were available: (1) the recommendation of the preceding version remains valid and requires no changes (“confirmed”); (2) the recommendation requires modification (“modified”); (3) the recommendation is no longer valid or required and is deleted; (4) a new recommendation needs to be developed (“new”). An interdisciplinary expert group of clinicians with expertise in trauma surgery, acute care, radiology and intensive care reviewed the body of evidence, drafted recommendations based on the homogeneity of clinical characteristics and outcomes, the balance between benefits and harms, as well as their clinical expertise, and proposed grades of recommendation (Table 2). In the absence of eligible evidence, good practice recommendations were made based on clinical experience, data from studies with a lower level of evidence, and expert consensus in cases where the Guideline Group felt a statement was required due to the importance of the topic. These were not graded, and instead labelled as good (clinical) practice points (GPP). For GPPs, the strength of a recommendation is presented in the wording shown in Table 2.

**Table 2:** Grading of recommendations

Symbol	Grade of recommendation	Description	Wording (examples)
⇑⇑	A	strong recommendation	“use …”, “do not use …”
⇑	B	recommendation	“should use …”, “should not use …”
⇔	0	open recommendation	“consider using …”, “… can be considered”

### Consensus process

The Guideline Group finalised the recommendations during web-based, structured consensus conferences on 14 February 2022 and 15 March 2022 via Zoom (Zoom, Version: 5.x [Software]. Zoom Video Communications, Inc., San José, California, USA. https://zoom.us). A neutral moderator facilitated the consensus conference. Voting members of the Guideline Group were delegates of all participating professional organisations, including clinicians, emergency medical services personnel and nurses, while guideline methodologists attended in a supporting role. Members with a moderate, thematically relevant conflict of interest abstained from voting on recommendations, members with a high, relevant conflict of interest were not permitted to vote or participate in the discussion. Attempts to recruit patient representatives were unsuccessful. A member of the expert group presented recommendations. Following discussion, the Guideline Group refined the wording of the recommendations and modified the grade of recommendation as needed. Agreement with both the wording and the grade of recommendation was assessed by anonymous online voting using the survey function of Zoom. Abstentions were subtracted from the denominator of the agreement rate. Consensus strength was classified as shown in Table 3.

**Table 3:** Classification of consensus strength

Description	Agreement rate
strong consensus	> 95% of participants
consensus	> 75 to 95% of participants
majority approval	> 50 to 75% of participants
no approval	< 50% of participants

Recommendations were accepted if they reached consensus or strong consensus. For consensus recommendations with ≤ 95% agreement, diverging views by members of the Guideline Group were detailed in the background texts. Recommendations with majority approval were returned to the expert group for revision and further discussion at a subsequent consensus conference. Recommendations without approval were considered rejected.

### External review

During a 4-week consultation phase, the recommendations and background texts were submitted to all participating professional organisations for review. Comments were collected using a structured review form. The results were then assessed, discussed and incorporated into the text by the guideline coordinator with the relevant author group.

The guideline was adopted by the executive board of the German Trauma Society on 17 January 2023.

### Quality assurance

The guideline recommendations were reviewed for consistency between guideline topic areas by the steering group. Where necessary, changes were made in collaboration with the clinical leads for all topic areas concerned. The final guideline document was checked for errors by the guideline chair and methodologist.

## Results

**Fig. 1:**
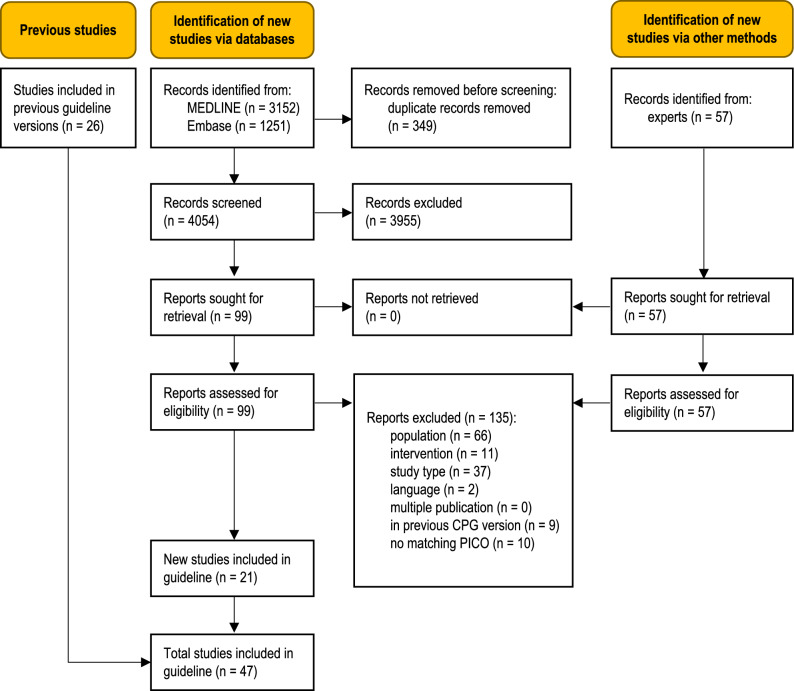
Modified PRISMA 2020 flow diagram showing the systematic literature search update and selection of studies

The database searches identified 4054 unique records (Fig. 1). Additional records were obtained from clinical experts. Twenty-one studies were eligible for this update [39–59]. A total of 135 full-text articles were excluded (Table S3, Online Resource 1).

### Characteristics of studies included in this update

Study characteristics, main outcomes, levels of evidence, and risk-of-bias assessments are presented in Table 4. Full details are provided in Table S4, Online Resource 1. The evidence included one RCT [54], one secondary analysis of an RCT [56], one prospective cohort study [44], five comparative registry studies [47, 49, 51, 55, 57], eleven diagnostic cross-sectional studies [39–41, 43, 45, 46, 48, 50, 52, 53, 59], one before-and-after study [58], and one case–control study [42]. Four studies were performed in the United States [40, 44, 52, 53], nine in Europe [42, 49–51, 54–56, 58, 59], five in the Middle East [39, 41, 45, 46, 48], two in Japan [47, 57], and one in Brazil [43]. Eligible patient populations were adults with severe injuries. Eleven studies included patients with abdominal and/or thoracic trauma [39–41, 43, 45, 46, 48, 50, 52, 53, 59].

**Table 4:** Characteristics of studies included in the update (see Table S4, Online Resource 1 for details)

Study, ref, design	Population	Interventions (N patients)	Main outcomes (selection)*	LoE, risk of bias (RoB)^§^, comments
*FAST for the detection of free fluid after blunt or penetrating abdominal trauma*				
Akdemir 2019 [39] Diagnostic cross-sectional study	Patients with blunt trauma	N = 315 Index test: FAST Reference test: contrast-enhanced CT	Detection of free fluid *Sensitivity, % (95% CI)* 82.3 (65.4–93.2) *Specificity, % (95% CI)* 100 (97.2–100)	LoE: 2b Unclear RoB
Akoglu 2017 [40] Diagnostic cross-sectional study	Patients with multiple trauma	N = 144 Index test: eFAST Reference test: contrast-enhanced CT	Detection of free fluid *Sensitivity, % (95% CI)* 42.9 (9.9, 81.6) *Specificity, % (95% CI)* 98.4 (94.3, 99.8)	LoE: 2b Unclear RoB
Bagheri-Hariri 2019 [41] Diagnostic cross-sectional study	Patients with blunt abdominal or chest trauma	N = 115 Index test 1: physical examination Index test 2: physical examination + eFAST Reference test: CT or intraoperative findings	**Index test 1** Detection of haemorrhagic shock *Sensitivity, % (95% CI)* 86.7 (59.5–98.3) *Specificity, % (95% CI)* 98.0 (93.0–99.8) Detection of haemoperitoneum *Sensitivity, % (95% CI)* 38.5 (13.9–68.4) *Specificity, % (95% CI)* 98.0 (93.1–99.8) Detection of solid organ damage *Sensitivity, % (95% CI)* 27.3 (6.0–61.0) *Specificity, % (95% CI)* 97.1 (91.8–99.4) **Index test 2** Detection of haemorrhagic shock *Sensitivity, % (95% CI)* 80.0 (51.9–95.7) *Specificity, % (95% CI)* 98.0 (93.0–99.8) Detection of haemoperitoneum *Sensitivity, % (95% CI)* 76.9 (46.2–95.0) *Specificity, % (95% CI)* 100 (96.5–100) Detection of solid organ damage *Sensitivity, % (95% CI)* 90.9 (58.7–99.8) *Specificity, % (95% CI)* 100 (96.5–100)	LoE: 2b Unclear RoB
Zanobetti 2018 [59] Diagnostic cross-sectional study	Trauma patients	N = 601 Index test: CA-FAST Reference test: thoraco-abdominal CT	Detection of free fluid *Sensitivity, % (95% CI)* 75 (67–83) *Specificity, % (95% CI)* 96 (93–97)	LoE: 3b Unclear RoB
*Detection of pneumothorax or haemothorax using transthoracic ultrasound, serial ultrasound examinations*				
Akoglu 2017 [40] Diagnostic cross-sectional study	Patients with multiple trauma	N = 144 Index test: eFAST Reference test: contrast-enhanced CT	Detection of pleural effusion *Sensitivity, % (95% CI)* 100.0 (15.8, 100.0) *Specificity, % (95% CI)* 100.0 (97.2, 100.0) Detection of pneumothorax *Sensitivity, % (95% CI)* 75.0 (35.0, 96.8) *Specificity, % (95% CI)* 99.2 (95.5, 100.0)	LoE: 2b Unclear RoB
Bagheri-Hariri 2019 [41] Diagnostic cross-sectional study	Patients with blunt abdominal or chest trauma	N = 115 Index test 1: physical examination Index test 2: physical examination + eFAST Reference test: CT or intraoperative findings	**Index test 1** Detection of pneumothorax *Sensitivity, % (95% CI)* 83.3 (35.9–99.6) *Specificity, % (95% CI)* 94.5 (88.4–97.9) Detection of haemothorax *Sensitivity, % (95% CI)* 20.0 (0.51–71.6) *Specificity, % (95% CI)* 99.1 (95.0–100) **Index test 2** Detection of pneumothorax *Sensitivity, % (95% CI)* 90.9 (58.7–99.8) *Specificity, % (95% CI)* 98.1 (93.2–99.8) Detection of haemothorax *Sensitivity, % (95% CI)* 80.0 (28.4–99.5) *Specificity, % (95% CI)* 100 (96.7–100)	LoE: 2b Unclear RoB
Ezzat 2018 [45] Diagnostic cross-sectional study	Patients with polytrauma	N = 80 Index test: X-ray and ultrasonography Reference test: whole-body CT	*Sensitivity, % (95% CI)* 90.32 (n.r.) *Specificity, % (95% CI)* 88.89 (n.r.)	LoE: 2b Unclear RoB
Kozaci 2019 [48] Diagnostic cross-sectional study	Trauma patients with thoracic injuries	N = 76 Index test: ultrasonography Reference test: chest CT	Detection of pneumothorax *Sensitivity [%] (95% CI)* 86 (n.r.) *Specificity [%] (95% CI)* 97 (n.r.) Detection of haemothorax *Sensitivity [%] (95% CI)* 45 (n.r.) *Specificity [%] (95% CI)* 98 (n.r.) Detection of pulmonary contusion *Sensitivity [%] (95% CI)* 63 (n.r.) *Specificity [%] (95% CI)* 91 (n.r.)	LoE: 2b High risk regarding flow and timing
Leblanc 2014 [50] Diagnostic cross-sectional study	Patients with multiple blunt trauma	N = 45 Index test 1: physical examination and x-ray Index test 2: ultrasonography Reference test: CT	**Index test 1** Detection of pneumothorax *Sensitivity [%] (95% CI)* 50 (n.r.) *Specificity [%] (95% CI)* 92 (n.r.) Detection of haemothorax *Sensitivity [%] (95% CI)* 52 (n.r.) *Specificity [%] (95% CI)* 80 (n.r.) Detection of pulmonary contusion *Sensitivity [%] (95% CI)* 78 (n.r.) *Specificity [%] (95% CI)* 57 (n.r.) **Index test 2** Detection of pneumothorax *Sensitivity [%] (95% CI)* 53 (n.r.) *Specificity [%] (95% CI)* 95 (n.r.) Detection of haemothorax *Sensitivity [%] (95% CI)* 60 (n.r.) *Specificity [%] (95% CI)* 99 (n.r.) Detection of pulmonary contusion *Sensitivity [%] (95% CI)* 90 (n.r.) *Specificity [%] (95% CI)* 87 (n.r.) **Index test 1 vs. index test 2** Detection of pneumothorax *AUC-ROC (95% CI)* 0.81 (0.50–1.00) vs. 0.74 (0.48–1.00), p = 0.24^§^ Detection of haemothorax *AUC-ROC (95% CI)* 0.84 (0.59–1.00) vs. 0.73 (0.51–1.00), p < 0.05 Detection of pulmonary contusion *AUC-ROC (95% CI)* 0.88 (0.76–1.00) vs. 0.69 (0.47–0.92), p < 0.05	LoE: 2b Low RoB
Ojaghi Haghighi 2014 [46] Diagnostic cross-sectional study	Patients with severe multiple trauma	N = 150 Index test 1: ultrasonography Index test 2: portable chest radiography Reference test: CT	**Index test 1** Detection of pneumothorax *Sensitivity [%] (95% CI)* 96.15 (n.r.) *Specificity [%] (95% CI)* 100 (n.r.) Detection of haemothorax *Sensitivity [%] (95% CI)* 82.97 (n.r.) *Specificity [%] (95% CI)* 98.05 (n.r.) **Index test 2** Detection of pneumothorax *Sensitivity [%] (95% CI)* 34.61 (n.r.) *Specificity [%] (95% CI)* 97.95 (n.r.) Detection of haemothorax *Sensitivity [%] (95% CI)* 25.53 (n.r.) *Specificity [%] (95% CI)* 95.14 **(**n.r.)	LoE: 2b Unclear RoB
Zanobetti 2018 [59] Diagnostic cross-sectional study	Trauma patients	N = 601 Index test: CA-FAST Reference test: thoraco-abdominal CT	Detection of pneumothorax *Sensitivity, % (95% CI)* 84 (77–89) *Specificity, % (95% CI)* 98 (96–99) Detection of pleural effusion *Sensitivity, % (95% CI)* 82 (74–88) *Specificity, % (95% CI)* 97 (95–98) Detection of pulmonary contusion *Sensitivity [%] (95% CI)* 59 (51–66) *Specificity [%] (95% CI)* 98 (96–99)	LoE: 3b Unclear RoB
*Chest X-ray as an alternative to immediate CT of the chest*				
Bolteho Finho 2015 [43] Diagnostic cross-sectional study	Patients with blunt trauma	N = 74 patients Index test: set of three examinations (chest X-ray, pelvic X-ray and FAST) Reference test: CT scan of torso or clinical observation during hospitalisation	Detection of significant injuries *Sensitivity, % (95% CI)* 90 (n.r.) *Specificity, % (95% CI)* 93 (n.r.)	LoE: 3b High RoB
*Timing and indications for whole-body CT, trauma-specific protocol*				
Bieler 2020 [42] Case–control study	Trauma patients with ISS ≥ 16	N = 1314 SV: survivor group NSV: non-survivor group	Factors associated with survival or non-survival *WBCT, n (%), p* SV: 597 (91.1) NSV: 565 (86.4), p = 0.006	LoE: 3b High risk associated with statistical analysis
Lang 2017 [49] Comparative registry study	Trauma patients with thoracic injuries	N = 16,545 patients IG: WBCT (n = 8559) CG: pre-WBCT (X-ray of cervical spine, chest and pelvis, often followed by focused CT) (N = 5002)	*Hospital mortality [%], mean (95% CI)* IG: 15.6 (14.9–16.4) CG: 15.5 (14.5–16.5)	LoE: 2b High risk of selection bias
Palm 2018 [51] Comparative registry study	Trauma patients with ISS ≥ 9	N = 16,928 IG: WBCT (N = 11,307) CG: pre-WBCT (X-ray, abdominal ultrasound, focused CT (N = 5621)	*RISC-II adjusted standardisation* *Mortality %* IG: 15.2 CG: 15.7	LoE: 2b Unclear RoB
Sierink 2016 [54] RCT	Patients with severe trauma^a^	N = 1083 IG: total-body CT (N = 541) CG: standard work-up (according to ATLS guideline) (N = 542)	*Inhospital mortality, n (%), p* IG: 86 (16%) vs. 85 (16%), p = 0.92	LoE: 1b High risk of performance bias
Topp 2015 [55] Comparative registry study	Trauma patients with ISS ≥ 16	N = 8020 IG: initial WB-MSCT (N = 4025) CG: conventional radiographs before WB-MSCT (N = 3995)	*RISC-II adjusted standardisation* *Mortality, SMR, p* IG: 0.86 CG: 0.85, p = 0.91	LoE: 2b Unclear RoB
CT scanner location				
Wulffeld 2017 [58] Retrospective before-and-after-study	Trauma patients	N = 1310 IG: mobile CT scanners with a moving gantry in the resuscitation room (N = 742) CG: before rebuilding (N = 784)	**Multivariate regression** *Mortality OR, (95% CI)* 1.1 (0.59–2.05)	LoE: 2b Unclear RoB
*Whole body CT imaging with contrast in haemodynamically unstable patients with severe injuries*				
Cook 2015 [44] Prospective cohort study	Trauma patients with hypotension and positive FAST exam^b^	N = 92 IG: positive FAST, hypotension and diagnostic CT (N = 32) CG: positive FAST, hypotension, no diagnostic CT (N = 60)	**Multivariate regression** *24-h mortality, OR (95% CI)* 0.41 (0.05–3.6)	LoE: 2b Unclear RoB
Katayama 2018 [47] Comparative registry study	Patients with blunt traumatic aortic injury in chest/abdomen	N = 421 IG1: time interval from hospital arrival to CT scanning 27–40 min; N = 135 IG2: time interval from hospital arrival to CT scanning > 41 min; N = 144 CG: time interval from hospital arrival to CT scanning < 26 min; N = 142	**Multivariate regression** *Death in the ED, OR (95% CI)* IG1: 1.833 (0.601–5.590), p = 0.287 IG2: 2.832 (1.007–7.960); p = 0.048	LoE: 2b Unclear RoB
Tsutsumi 2017 [57] Comparative registry study	Patients with blunt trauma and SBP < 90 mmHg	N = 5809 IG: CT (N = 5352) CG: no CT (N = 457)	**Multivariate regression** *Inhospital mortality, n (%), IPTW (PS) per 100 patients (95% CI)* IG: 655 (12.8) CG: 147 (34.9), p < 0.001 − 20.6 (− 26.2 to − 14.9)	LoE: 2b Unclear RoB
*Prehospital ultrasound*				
Press 2014 [52] Diagnostic cross-sectional study	Trauma patients	N = 833 Patients with at least one HEMS ultrasound exam (N = 293) Number of lung HEMS ultrasound exams (N = 511) Index test: in-flight ultrasound Reference standard: diagnostic procedures and management in the ED including CT, chest radiography and clinical examination	Detection of pneumothorax *Sensitivity, % (95% CI)* 18.7 (8.9–33.9) *Specificity, % (95% CI)* 99.5 (98.2–99.9)	LoE: 2b Low RoB
Quick 2016 [53] Diagnostic cross-sectional study	Trauma patients	N = 149 patients receiving in-flight ultrasound Index test: in-flight ultrasound (N = 149) Reference standard: CT scan (N = 116)	Detection of pneumothorax *Sensitivity, % (95% CI)* 68 (0.46–0.85) *Specificity, % (95% CI)* 96% (CI 0.90–0.98)	LoE: 2b Unclear RoB
*Whole-body CT*				
Treskes 2020 [56] Secondary analysis of an RCT	Trauma patients^a^	N = 1083 Study groups/original REACT-2 iTBCT criteria, for details see Table S4, Online Resource 1	*Number needed to overscan†, n (95% CI)* 5.6 (4.9–6.5)	LoE: 2b Unclear RoB

*adj*. adjusted, *ATLS* Advanced Trauma Life Support, *AUC-ROC* area under the curve-receiver operating characteristics, *CG* control group, *CT* computed tomography, *d* days, *ED* emergency department, *eFAST* extended focused assessment with sonography for trauma, *FAST* focused assessment with sonography for trauma, *h* hours, *HEMS* Helicopter Emergency Medical Services, *IG* intervention group, *IPTW (PS)* inverse probability of treatment weighted analysis based on propensity score, *ISS* Injury Severity Score, *n.r*. not reported, *n.s*. not significant, *OR* odds ratio, *RISC-II* Revised Injury Severity Classification 2, *RCT* randomised controlled trial, *SBP* systolic blood pressure, *SMR* standardised mortality rate, *WBCT* whole-body computed tomography, *WB-MSCT* whole-body multi-slice computed tomography, *y* years

*Data for IG versus CG unless otherwise specified

^§^Risk of bias: low RoB = RoB low for all domains; unclear RoB = RoB unclear for at least one domain, no high RoB in any domain; for studies with high RoB, all domains with high RoB are named, with RoB low or unclear for all other domains (for full details Table S4, Online Resource 1)

^a^Presence of life-threatening vital problems (at least one of the following: respiratory rate ≥ 30 min of ≤ 10/min; pulse ≥ 120/min; systolic blood pressure ≤ 100 mmHg; estimated exterior blood loss ≥ 500 mL; Glasgow Coma Score ≤ 13; abnormal pupillary reaction onsite, clinically suspicious diagnoses), (flail chest, open chest or multiple rib fractures; severe abdominal injury; pelvic fracture; unstable vertebral fractures/spinal cord compression; fractures from at least two long bones), injury mechanisms (fall from height > 3 m / > 10 ft); ejection from the vehicle; death occupant in same vehicle; severely injured patient in same vehicle; wedged or trapped chest/abdomen)

^b^Received one or more units of red blood cells (RBCs) within 6 h of hospital admission; hypotension defined as an admission systolic blood pressure (SBP) ≤ 90 mmHg

### Risk-of-bias assessment for included studies and levels of evidence

Two studies were judged to be of low risk of bias in all domains. The risk of selection bias was high in two studies and unclear in seven studies. The risk of bias regarding the index and reference tests was high in one study and unclear in two and four studies, respectively. The risk of bias regarding flow and timing was high in one study and unclear in four studies. The risk of performance bias was high in one study and unclear in six studies. In one study, statistical analysis may have led to a bias.

### Recommendations

Six recommendations were modified. One additional good practice point was developed based on the updated evidence and expert consensus (Table 5). All except one achieved strong consensus. Three recommendations from the 2016 Guideline were not retained in the 2022 update (Table S5, Online Resource 1).

**Table 5:** List of recommendations with grade of recommendation and strength of consensus

No	GoR	Evidence, consensus^a^	Recommendation	Status 2022
*Sonography/ultrasound*				
1	B ⇑	[39–41, 45, 48, 50, 59] 100%	Extended focused assessment with sonography for trauma (eFAST) should be performed for diagnostic purposes after blunt and/or penetrating thoracic and/or abdominal trauma as part of the primary survey in the resuscitation room	Modified
2	B ⇑	[39–41, 45, 48, 50, 59–65] 100%	Serial ultrasound examinations of the chest and/or abdomen should be performed to evaluate patients with pathological findings after whole-body computed tomography (WBCT)	Modified
*Chest and pelvic X-rays*				
3	B ⇑	[43, 66–69] 100%	If it remains unclear whether or not a relevant thoracic injury is present and immediate computed tomography of the chest cannot be performed, a chest X-ray should be taken	Modified
4	GPP	100%	If it remains unclear whether or not a relevant pelvic injury is present and immediate computed tomography cannot be performed, a pelvic X-ray may be taken	Modified
*Computed tomography (CT)/whole-body computed tomography (WBCT)*				
5	A ⇑⇑	[42, 47, 49, 51, 54, 55, 57, 70–73] 100%	As part of the diagnostic procedures for severely injured patients, perform whole-body computed tomography* with a trauma-specific protocol in a timely manner if the patient does not require an immediate intervention, a surgical procedure or resuscitation and if systolic blood pressure is not lower than 60 mmHg *(head-to-pelvis WBCT, cranial computed tomography without contrast)	Modified
6	B ⇑	[56] 100%	Whole-body CT should be performed in patients with suspected severe and/or multiple injuries and Compromised vital parameters (circulation, breathing, consciousness, neurological function) Pathological findings on clinical examination and/or imaging of the chest and/or abdomen and/or pelvis and/or spine Fractures of at least two long bones A relevant mechanism of injury (fall from a height of more than four metres, trapped chest/abdomen)	Modified
*Magnetic resonance imaging (MRI)*				
7	GPP	85.7%	Magnetic resonance imaging (MRI) can be indicated as a further primary diagnostic tool for specific conditions (e.g. discoligamentous injuries, morphological correlates of symptoms of spinal cord injury). The use of MRI as an initial diagnostic imaging modality for patients with severe and/or multiple injuries must meet extensive requirements. These requirements should be defined in local standard operating procedures (SOPs)	New

*GoR* grade of recommendation

## Discussion

### Rationale for recommendations

#### Sonography/ultrasound

An ultrasound examination of the abdomen, pericardium and pleura is an effective method of evaluating patients with actual or potential severe injuries in the emergency setting. The recommendations are based predominantly on studies with LoE 2b and an unclear or low risk of bias. Regarding the flow and timing of the ultrasound examination, the risk of bias was classified as high.

The Focused Assessment with Sonography for Trauma (FAST) examination is a tool for the evaluation of the abdomen. The extended FAST is used to additionally evaluate the chest and the pleura. According to the 2021 Annual Report of the TraumaRegister^®^ of the German Trauma Society, eFAST is performed in approximately 82% of patients as part of the primary survey in the resuscitation room setting [21].

Sonography is an imaging modality that provides cross-sectional images. It shows varying levels of sensitivity and can be highly specific. Examiners require a high level of experience. Examination results are difficult to document and reproduce. Ultrasound is not as valuable a guide to decision making as CT [46, 74–77]. The literature reports that ultrasound is associated with a high level of specificity (94–100%) and varying levels of sensitivity (28–100%) [60–65].

Compared with the 2016 Guideline [78], a number of studies have been additionally included in the updated version.

Akdemir et al. conducted an analysis that involved 315 patients and found that ultrasound had a sensitivity of 82.3% and a specificity of 100% for detecting free fluid after blunt trauma [39]. Akoglu et al. found that ultrasound had a sensitivity of 42.9% and a specificity of 98.4% for the detection of free fluid, a sensitivity and a specificity of 100% for haemothorax, and a sensitivity of 75% and a specificity of 99.2% for pneumothorax [40]. Similar results were reported in other studies [41, 45, 48, 50, 59, 76].

All of the aforementioned authors emphasised that a negative eFAST did not exclude an intra-abdominal or intrathoracic injury. The higher the overall injury severity score, the less accurate eFAST can be. In these cases, a repeat ultrasound examination or a CT scan should be additionally performed [39–41, 45, 48, 50, 59–65, 79].

#### Chest and pelvic X-rays

There is a paucity of literature-based evidence on the diagnostic accuracy of conventional anteroposterior (AP) chest radiography in the management of severely injured patients. The recommendation for chest X-ray is based on a LoE 3b study with a high potential for bias.

Wilkerson and Stone [68] conducted a systematic review of the literature and identified four relevant studies [66, 67, 69, 80] (n = 606 patients). They found that AP chest radiographs had a sensitivity ranging from 28 to 76% for detecting pneumothorax and a specificity of 100%. A further study was included in the updated version of the Guideline. Botelho et al. reported a sensitivity of 90% and a specificity of 93% for identifying significant injuries [43].

There is no evidence on the role of pelvic radiographs in the management of severely injured patients which meets the inclusion criteria for an S3 guideline. For this reason, the recommendation in the 2016 Guideline was modified at the expert level and the word “should” was replaced by “may”.

In clinical practice, the anteroposterior WBCT scout image (e.g. of the chest or pelvis) can be used to detect significant pneumothoraces or haemothoraces and/or pelvic fractures. The absence of scout view findings, however, does not exclude the presence of such conditions.

#### Computed tomography (CT)/whole-body computed tomography (WBCT)

The two key recommendations in the 2016 Guideline [78] on WBCT in general and on the use of WBCT in haemodynamically unstable patients have been united into a single key recommendation (Table 5). The recommendations are based on studies of LoE 1b to 3b and mostly have an unclear risk of bias. A study of LoE 1b has a high risk of bias with regard to performance, a study of LoE 2b has a high risk of selection bias and a study of LoE 3b has a high risk of statistical analysis bias.

The first part of the key recommendation, which addresses the *general* use of WBCT, is based on the following evidence.

In 2009, Huber-Wagner, Lefering and collaborators conducted a multi-centre analysis of 4621 patients from the TraumaRegister^®^ of the German Trauma Society and showed for the first time that the use of WBCT in the resuscitation room setting significantly increased the probability of survival. The analysis was adjusted for severity. On the basis of the revised injury severity classification (RISC) prognostic scores, the predicted mortality rate was 23% and the observed mortality rate was 20% [81, 82]. This corresponds to a “number needed to scan” of 32. In other words, every thirty-second severely injured patient who undergoes WBCT will survive contrary to the patient’s prognosis [72].

In another analysis of a total of 16,719 patients from the TraumaRegister^®^ of the German Trauma Society, Huber-Wagner et al. found that severely injured patients who underwent initial WBCT had an absolute mortality rate of 17.4%. This group of patients was compared with patients who did not undergo WBCT. Their mortality rate was 21.4% (p < 0.001) [73].

Based on an analysis of 4814 patients, Kanz et al. also reported a significant increase in the probability of survival of patients who underwent WBCT [70].

In 2012, Stengel et al. conducted a study on 982 patients and found that WBCT had a sensitivity of 85–92%, a specificity of 95–99%, a positive predictive value of 95–99%, and a negative predictive value of 86–97% in severely injured patients [71]. The authors thus demonstrated high diagnostic accuracy of WBCT. Compared with other radiological procedures, WBCT has the highest diagnostic accuracy and reliability [71].

In 2016, Sierink et al. published their Randomised Study of Early Access by CT Scanning (REACT-2) trial. In this randomised controlled study, they compared 541 patients who underwent WBCT and 542 patients who underwent conventional imaging and selective CT. The authors did not find a significant survival benefit from WBCT [54]. There are, however, relevant limitations in study design. Only two thirds of patients had an Injury Severity Score (ISS) > 16. As a result of overlapping protocols, 73% of the 1083 patients rather than the 50% mentioned in the study underwent WBCT. The number of severely injured patients (with an ISS > 16) was too low to reach statistical significance (p < 0.05) (mortality rate of 22% versus 25%, WBCT versus standard work-up, p = 0.46, Table 2 of the study). As a result of a suboptimal study protocol, the ambitious study by Sierink et al. does not change the existing evidence in support of WBCT [83].

Other studies, some of which are based on analyses of data from the TraumaRegister^®^ of the German Trauma Society [42, 49, 55], have become available recently [47, 51, 54]. In addition, several meta-analyses and systematic reviews confirm the positive effect of WBCT on survival [28, 84–88].

The second part of the key recommendation, which addresses *the use of WBCT in haemodynamically unstable patients in shock*, is based on the following evidence.

In an analysis of 16,719 patients from the TraumaRegister® of the German Trauma Society, Huber-Wagner et al. were able to show that WBCT was also useful in haemodynamically compromised trauma patients who were in a pre-shock state. The standardised mortality ratio (SMR) for patients in severe shock (with a systolic blood pressure < 90 mmHg at hospital admission) was 42.1% for those who underwent WBCT and 54.9% for those who did not undergo WBCT (p < 0.001). The authors pointed out that especially patients in a pre-shock state might benefit from WBCT since this imaging modality can rapidly and comprehensively detect the cause(s) of shock. It should be noted that patients with manifest circulatory failure who died within the first thirty minutes of arrival at the hospital were excluded because of an “immortal time bias” [73].

Tsutsumi et al. analysed data from the Japanese Trauma Data Bank and reported similar results for a total of 5809 haemodynamically unstable patients with an admission SBP < 90 mmHg and > 40 mmHg. Both an analysis of raw data and an analysis that was adjusted for confounding factors showed that the inhospital mortality rate was significantly lower in patients who underwent CT (23.8%) than in patients without CT (45.3%, p < 0.001) [57].

In a study on 92 patients with a positive eFAST, Cook et al. compared patients who underwent abdominal CT after trauma with those who did not and found no difference in mortality between the two groups but a lower rate of emergency surgery in the group of patients who had a CT [44].

These findings suggest that the information obtained from WBCT can impact treatment decisions (e.g. evidence for or against emergency surgery) also in haemodynamically compromised patients (with an SBP between 60 and 90 mmHg) [44, 73]. During WBCT, patients must have effective circulation (the flow of contrast must be ensured). Further requirements are a trained trauma team and appropriate infrastructure [73]. Severely unstable patients with an admission SBP < 60 mmHg, patients in extremis, and patients undergoing resuscitation often require and can benefit from a stabilising emergency operation or intervention prior to diagnostic CT. WBCT should not be performed in patients undergoing resuscitation (massive motion artefacts, unstable flow of contrast, radiation exposure of personnel, etc.).

Full-body linear X-ray scanning in polytrauma patients cannot replace WBCT. Whole-body X-ray examinations are useful only in patients with injuries to the extremities. Such techniques have an overall sensitivity of < 50% for injuries to the skeleton of the trunk [89].

The *indications* for WBCT in patients with suspected multiple and/or severe injuries are based on the following evidence.

Davies et al. analysed data from 255 patients and developed a scoring system that can be used as a decision tool. They recommended the use of WBCT for patients with a score > 3 and the selective use of CT for patients with a score ≤ 3. Injuries to two or more regions were given a score of + 2, haemodynamic instability a score of + 2, respiratory abnormality a score of + 3, a Glasgow Coma Scale (GCS) score < 14 a score of + 3, a fall from ≤ 5 m a score of − 1, involvement in a road accident as a driver or passenger a score of + 1, involvement in a road accident as a cyclist or pedestrian a score of + 3, and a fall from > 5 m a score of + 3 [90].

Hsiao et al. conducted a study in which they used logistic regression and found that especially patients with multi-region injuries would benefit from WBCT. Predictors of multi-region injuries or polytrauma were GCS < 9, haemodynamic instability, falls from a height of more than five metres, and involvement in a road accident as a cyclist [91].

Huber-Wagner et al. too developed a decision tool, i.e. the whole-body CT score, on the basis of an analysis of data from 78,180 patients from the TraumaRegister^®^ of the German Trauma Society. For this purpose, they performed a propensity score analysis. A score between 0 and 3 is indicative of a moderate benefit of WBCT. A score between − 16 and − 1 means that WBCT has no survival benefit for patients undergoing WBCT, a score between 4 and 16 suggests a survival benefit, and a score between 17 and 35 indicates a great survival benefit. The following scores were assigned: intubation at the scene of an accident (+ 8), suspicion of injury to ≥ 3 body regions (+ 8), high-energy trauma (+ 7), air medical transport (+ 5), GCS ≤ 14 (+ 3), suspicion of injury to two body regions (+ 3), presence of shock at the scene (+ 2), male gender (+ 2), penetrating trauma (− 7), fall from a height < 3 m (− 7), age < 70 years (− 1), suspicion of injury to one body region (− 1) [92].

In 2022, Treskes et al. published a study that identified the following criteria for WBCT on the basis of data from the REACT-2 study: systolic blood pressure < 100 mmHg, estimated blood loss ≥ 500 mL, GCS ≤ 13, fractures of at least two long bones, flail chest, open chest injury, multiple rib fractures, severe abdominal or pelvic injury, unstable vertebral fractures/spinal cord compression, fall from a height of more than four metres [56].

These studies define the key criteria or predictors for WBCT, i.e. compromised vital signs, relevant mechanism of injury, *and* the presence of injuries to more than one body region [91].

This also means that WBCT should only be performed in patients with suspected polytrauma that meets the definition in the current guideline. The mechanism of injury alone, i.e. without *clinical* evidence of injuries, is not a sufficient indication for WBCT.

Clinically stable patients with only one injured non-torso body region (no polytrauma) can be appropriately managed with a staged approach consisting of eFAST and/or standard ultrasound, radiographic examinations of the clinically involved body regions, and—where appropriate—selective-organ CT scans, as well as clinical and imaging follow-up depending on the clinical course [93].

#### Whole-body CT: special aspects

Particular attention is drawn to the revised Guideline of the German Medical Association on Quality Assurance in Computed Tomography and Diagnostic Radiographic Examinations (QA Guideline) of 2022, which newly includes a section on polytrauma and whole-body CT. The QA guideline systematically defines all CT scanner requirements and technical aspects of CT examinations (e.g. time-optimised versus dose-optimised protocols) and provides direction for clinical practice [1]. Compliance with the QA Guideline of the German Medical Association on the use of WBCT in polytrauma patients is supervised by Medical Bodies, which are established by the Regional Medical Associations and provide relevant advice. The recommendations in the QA Guideline are rated as strong.

Another relevant guideline is the European Society of Emergency Radiology (ESER) Guideline on Radiological Polytrauma Imaging and Service, which was published in 2020. This comprehensive and detailed guideline addresses essential aspects of diagnostic imaging in the management of severely injured patients. It was published in two versions: a full version and a short version [76]. Particular care must be taken to ensure that WBCT with contrast allows the arterial vascular system and parenchymal organs to be evaluated in a single scan in order to detect relevant bleeding. Further details are provided in the literature [1, 76, 94–98]. Moreover, the Working Group on Musculoskeletal Imaging (AG MSK) of the German Radiological Society recommends standard imaging protocols on its website in an effort to standardise WBCT imaging nationwide. These protocols incorporate recommendations provided in the QA Guideline of the German Medical Association.

Furthermore, due to the continuing lack of evidence, further studies are necessary. Two major areas can be identified. Firstly, the device technology must be optimized in order to keep the radiation exposure for patients as low as possible. Secondly, prospective studies must be carried out that focus on which patients benefit from WBCT in terms of mortality and functional outcome based on which physiological parameters and with which injury patterns and injury mechanisms, taking into account patient-specific factors.

#### Magnetic resonance imaging

Magnetic resonance imaging (MRI) can be indicated as a further primary diagnostic tool (for example within 12–24 h of trauma) for specific conditions (e.g. discoligamentous injuries, morphological correlates of symptoms of spinal cord injury, brainstem injuries). The use of MRI as an initial diagnostic imaging modality for patients with severe and/or multiple injuries must meet extensive clinical, technical and organisational requirements. These requirements should be defined in local standard operating procedures (SOPs) [99–107].

### Limitations of the guideline

Patient values and preferences were sought but not received. The effect of this on the guideline is unclear, and there is a lack of research evidence on the effect of patient participation on treatment decisions or outcomes in the emergency setting.

## Supplementary Information

Below is the link to the electronic supplementary material.

Supplementary file1 (DOCX 127 KB)

## Data Availability

No datasets were generated or analysed during the current study.

## Abbreviations

AG MSK: Working Group on Musculoskeletal Imaging
AWMF: Association of the Scientific Medical Societies in Germany
CCT: Cranial computed tomography
CI: Confidence interval
CT: Computed tomography
eFAST: Extended focused assessment with sonography for trauma
ESER: European Society of Emergency Radiology
FAST: Focused assessment with sonography for trauma
GCS: Glasgow Coma Scale
GPP: Good (clinical) practice point
GoR: Grade of recommendation
IFOM: Institute for Research in Operative Medicine
ISS: Injury Severity Score
LoE: Level of evidence
MRI: Magnetic resonance imaging
NICE: National Institute for Health and Care Excellence
PACS: Picture archiving and communication system
PICO: Population, intervention, comparison, outcome
PRISMA: Preferred reporting items for systematic reviews and meta-analyses
RCT: Randomised controlled trial
REACT: Randomised study of early access by CT scanning
RIS: Radiology information system
RISC: Revised injury severity classification
QA: Quality assurance
SBP: Systolic blood pressure
SMR: Standardised mortality ratio
SOP: Standard operating procedure
TRISS: Trauma and Injury Severity Score
WBCT: Whole-body computed tomography
