# Supplementary material for: Imaging strategies for patients with multiple and/or severe injuries in the resuscitation room: a systematic review and clinical practice guideline update
Source: Eur J Trauma Emerg Surg. 2025 Apr 2;51(1):158. doi: 10.1007/s00068-025-02840-8 (PMC11965254; doi:10.1007/s00068-025-02840-8)
Supplement: Supplementary file 1 — Supplementary file1 (DOCX 127 KB) [file 68_2025_2840_MOESM1_ESM.docx]

2.18 Imaging

# S1 PICO Questions

|  | Population | Intervention(s) | Control(s) | Outcome(s) |
| --- | --- | --- | --- | --- |
| 1 | Target population, with blunt or penetrating abdominal trauma. | eFAST* as part of the Primary Survey | CT, X-ray, other | Patient-relevant outcomes, DTA |
| 2 | Target population, without prompt CT scan | Repeat sonographic examinations | Singular sonographic examination | Patient-relevant outcomes, DTA |
| 3 | Target population, with mainly pneumothorax or haematothorax | transthoracic ultrasound examination | Other, CT, etc. | Patient-relevant outcomes, DTA |
| 4 | Target population, with unclear thoracic injury, without prompt CT scan | X-ray of the thorax | Other procedures, none | Patient-relevant outcomes, DTA |
| 5 | Target population, with unclear pelvic injury, without prompt CT scan | X-ray of the pelvic region | Other procedures, none | Patient-relevant outcomes, DTA |
| 6 | Target population, with a history of relevant injuries to the thorax, abdomen, pelvis or fractures of the spine or large tubular bones. | Full-body CT with trauma-specific protocol | Other procedures, selective CT, none | Patient-relevant outcomes, DTA |
| 7 | Target population, with a disturbance of vital parameters (circulation, respiration, consciousness), specific accident mechanism, at least 2 relevant injured body regions | Full body CT | Other procedures, selective CT, none | Patient-relevant outcomes, DTA |
| 8 | Target population | CT close to the shock room | CT further away from shock room | Patient-relevant outcomes |
| 9 | Target population, haemodynamically unstable | Whole-body CT with contrast medium | Other procedures, no contrast medium, none | Patient-relevant outcomes, DTA |
| 10^§^ | Target population, with specific indications | CT | No CT/ other procedure | Patient-relevant outcomes, DTA |
| 11^§^ | Target population, with specific indications | Prehospital sonography | No (later) sonography / other procedure | Patient-relevant outcomes, DTA |
| 12^§^ | Target population, with specific indications | Whole-body CT | No whole-body CT / other procedure | Patient-relevant outcomes, DTA |
| 13^§^ | Target population, with initially unremarkable portion of abdominal CT | Re-Fast | No re-fast / singular e-fast / other procedure | Patient-relevant outcomes, DTA |
| 14^§^ | Target population, with specific indications | MRI | No MRI / other procedure | Patient-relevant outcomes, DTA |
| 15^§^ | Target population | Prehospital invasive emergency technique (e.g. relief of pneumothorax, pericardial tamponade) with sonography | Non-invasive emergency technique/ Invasive emergency technique without sonography | Patient-relevant outcomes |

# S2 Literature Search

| Suchstrategie 2021, MEDLINE (via Ovid) Datum: 27.08.2021 3.152 Treffer |
| --- |
| 1. exp Whole Body Imaging/ or *Radiography, Abdominal/ or *Radiography/ or *Radiography, Thoracic/ or *Tomography/ or *Tomography Scanners, X-Ray Computed/ or *Tomography, X-Ray Computed/ or *Tomography, Spiral Computed/ or *Tomography, X-Ray/ or *Magnetic Resonance Imaging/ or *Ultrasonography, Doppler/ or *Ultrasonography/ or *Ultrasonography, Doppler, Duplex/ or *Ultrasonography, Doppler, Color/ or *Focused Assessment with Sonography for Trauma/ or exp "Wounds and Injuries"/dg  2. ((whole body adj (imag* or scan* or CT or MR* or NMR or tomograph* or comput* tomograph*)) or radiograph* or x-ray or diagnostic x-ray or ((chest or thora* or abdom* or pelvic) adj radiograph*) or radar technology or comput* tomograph* or ct or ((ct or cat) adj scan*) or mdct or ((chest or thora* or abdom* or pelvic or spiral) adj ct) or magnetic resonance imag* or mrt or ((mr or nmr) adj tomograph*) or sonograph* or ultrasonograph* or ultrasound or focused assessment with sonography for trauma or Fast or eFast or ((cranial or spine) adj (ct or mr*)) or cct).ti,ab,kf.  3. 1 or 2  4. *Trauma Centers/ or (trauma cent* or trauma room or shock room or resuscitation area? or resuscitation room or (initial and (treatment or diagnos*)) or early phase or bedside or bed-side or hand-carried).ti,ab,kf. or (emergency or emergencies or emergent).ti. or (emergency or emergencies or emergent).ab. /freq=2  5. 3 and 4  6. exp Multiple Trauma/  7. (polytrauma* or trauma patient?).ti,ab,kf. or (severe adj2 shock).ti,ab,kf.  8. ((multiple or major or severe* or serious*) adj3 (trauma* or injur*)).ti,ab,kf.  9. ((blunt or penetrating) adj5 (trauma* or injur*)).ti,ab,kf.  10. (*Critical Care/ or *Emergencies/ or (life threatening or critical care or emergen*).ti,ab,kf.) and (trauma* or injur*).ti,ab,kf.  11. 6 or 7 or 8 or 9 or 10  12. 5 and 11  13. exp animals/ not humans.sh.  14. 12 not 13  15. (comment or editorial or letter).pt. or case report*.mp.  16. 14 not 15  17. limit 16 to dt=20140101-20210831 |
| Suchstrategie 2021, Embase (via Elsevier) Datum: 27.08.2021 1.251 Treffer |
| #1 'whole body imaging'/exp OR 'abdominal radiography'/mj OR 'radiography'/mj OR 'thorax radiography'/mj OR 'tomography'/mj OR 'computed tomography scanner'/mj OR 'x-ray computed tomography'/mj OR 'spiral computer assisted tomography'/mj OR 'x-ray tomography'/mj OR 'nuclear magnetic resonance imaging'/mj OR 'Doppler ultrasonography'/mj OR 'echography'/mj OR 'duplex Doppler ultrasonography'/mj OR 'color Doppler flowmetry'/mj OR 'focused assessment with sonography for trauma'/mj  #2 (("whole body" NEXT/1 (imag* OR scan* OR CT OR MR* OR NMR OR tomograph* OR "comput* tomograph*")) OR radiograph* OR x-ray OR "diagnostic x-ray" OR ((chest OR thora* OR abdom* OR pelvic) NEXT/1 radiograph*) OR "radar technology" OR "comput* tomograph*" OR ct OR ((ct OR cat) NEXT/1 scan*) OR mdct OR ((chest OR thora* OR abdom* OR pelvic OR spiral) NEXT/1 ct) OR "magnetic resonance imag*" OR mrt OR ((mr OR nmr) NEXT/1 tomograph*) OR sonograph* OR ultrasonograph* OR ultrasound OR "focused assessment with sonography for trauma" OR Fast OR eFast OR ((cranial OR spine) NEXT/1 (ct OR mr*)) OR cct):ti,ab,kw  #3 #1 OR #2  #4 'hospital emergency service'/mj OR ("trauma cent*" OR emergency OR emergencies OR emergent OR "trauma room" OR "shock room" OR "resuscitation area?" OR "resuscitation room" OR (initial AND (treatment OR diagnos*)) OR "early phase" OR bedside OR bed-side OR hand-carried):ti,ab,kw  #5 #3 AND #4  #6 'multiple trauma'/exp  #7 (polytrauma* OR "trauma patient?"):ti,ab,kw OR (severe NEXT/2 shock):ti,ab,kw  #8 ((multiple OR major OR severe* OR serious*) NEXT/3 (trauma* OR injur*)):ti,ab,kw  #9 ((blunt OR penetrating) NEXT/5 (trauma* OR injur*)):ti,ab,kw  #10 ('intensive care'/mj OR 'emergency'/mj OR ("life threatening" OR "critical care" OR emergen*):ti,ab,kw) AND (trauma* OR injur*):ti,ab,kw  #11 #6 OR #7 OR #8 OR #9 OR #10  #12 #5 AND #11  #13 'animals'/exp NOT 'humans'/de  #14 #12 NOT #13  #15 (comment OR editorial OR letter):it OR "case report*":ti,ab,kw  #16 #14 NOT #15  #17 [1-1-2014]/sd NOT [31-8-2021]/sd  #18 #16 AND #17  #19 [embase]/lim  #20 #18 AND #19  #21 embase NOT (embase AND medline)  #22 #20 AND #21  #23 #22 AND ('article'/it OR 'article in press'/it OR 'erratum'/it OR 'review'/it) |

# S3 Excluded Studies

| **Reference** | **Reason for exclusion** |
| --- | --- |
| Abbasi, S., et al., *Screening performance of ultrasonographic b-lines in detection of lung contusion following blunt trauma; a diagnostic accuracy study.* Emergency, 2018. **6**(1). | Population |
| Abdulrahman, Y., et al., *Utility of extended FAST in blunt chest trauma: is it the time to be used in the ATLS algorithm?* World Journal of Surgery, 2015. **39**(1): p. 172-8. | Population |
| Abedi Khorasgani, M., et al., *The Accuracy of Plain Radiography in Detection of Traumatic Intrathoracic Injuries.* Emergency (Tehran, Iran), 2016. **4**(4): p. 184-187. | Population |
| Alabousi, M., N. Zha, and M.N. Patlas, *Use of Enteric Contrast for Abdominopelvic CT in Penetrating Traumatic Injury in Adults: Comparison of Diagnostic Accuracy Systematic Review and Meta-Analysis.* AJR. American Journal of Roentgenology, 2020. **30**: p. 30. | Intervention |
| Arhami Dolatabadi, A., et al., *Comparison of the accuracy and reproducibility of focused abdominal sonography for trauma performed by emergency medicine and radiology residents.* Ultrasound in Medicine & Biology, 2014. **40**(7): p. 1476-82. | Intervention |
| Arruzza, E., M. Chau, and J. Dizon, *Systematic review and meta-analysis of whole-body computed tomography compared to conventional radiological procedures of trauma patients.* European Journal of Radiology, 2020. **129**: p. 109099. | Population |
| Aziz, A.A.E.R.A.E., et al., *Validity of the fast scan for diagnosis of intra-abdominal injury in blunt abdominal trauma.* Indian Journal of Public Health Research and Development, 2020. **11**(2): p. 1173-1179. | Population |
| Basnet, S., et al., *Diagnostic performance of the extended focused assessment with sonography for trauma (EFAST) patients in a tertiary care hospital of Nepal.* Trauma Surgery & Acute Care Open, 2020. **5**(1): p. e000438. | Population |
| Bolt, C., et al., *Straight leg elevation to rule out pelvic injury.* Injury, 2018. **49**(2): p. 279-283. | Population |
| Boutros, S.M., M.A. Nassef, and A.F. Abdel-Ghany, *Blunt abdominal trauma: The role of focused abdominal sonography in assessment of organ injury and reducing the need for CT.* Alexandria Journal of Medicine, 2016. **52**(1): p. 35-41. | Population |
| Bouzat, P., et al., *Early management of severe abdominal trauma.* Anaesthesia Critical Care & Pain Medicine, 2020. **39**(2): p. 269-277. | Study type |
| Chan, K.K., et al., *Chest ultrasonography versus supine chest radiography for diagnosis of pneumothorax in trauma patients in the emergency department.* Cochrane Database of Systematic Reviews, 2020. **7**: p. CD013031. | In previous CPG version |
| Dabees, N.L., et al., *Multi-detector computed tomography imaging of blunt chest trauma.* Egyptian Journal of Radiology and Nuclear Medicine, 2014. **45**(4): p. 1105-1113. | Population |
| Davoodabadi, A., et al., *Diagnostic value of serial ultrasound in blunt abdominal trauma.* Archives of Trauma Research, 2017. **6**(2). | Population |
| Elbaih, A.H. and S.T. Abu-Elela, *Predictive value of focused assessment with sonography for trauma (FAST) for laparotomy in unstable polytrauma Egyptians patients.* Chinese Journal of Traumatology, 2017. **20**(6): p. 323-328. | Population |
| Elfiky, I., et al., *Diagnostic performance of multi-slice computed tomography using 2D and 3D images in the assessment of Le Fort fractures.* Egyptian Journal of Radiology and Nuclear Medicine, 2017. **48**(2): p. 415-424. | Population |
| Engles, S., N.S. Saini, and S. Rathore, *Emergency Focused Assessment with Sonography in Blunt Trauma Abdomen.* International Journal of Applied & Basic Medical Research, 2019. **9**(4): p. 193-196. | Population |
| Feyzi, A., et al., *Diagnostic accuracy of ultrasonography in detection of blunt abdominal trauma and comparison of early and late ultrasonography 24 hours after trauma.* Pakistan Journal of Medical Sciences, 2015. **31**(4): p. 980-3. | Population |
| Frellesen, C., et al., *Evaluation of a dual-room sliding gantry CT concept for workflow optimisation in polytrauma and regular in- and outpatient management.* European Journal of Radiology, 2015. **84**(1): p. 117-122. | Population |
| Furugori, S., et al., *Treating patients in a trauma room equipped with computed tomography and patients' mortality: a non-controlled comparison study.* World Journal Of Emergency Surgery, 2018. **13**: p. 16. | Population |
| Ghafouri, H.B., et al., *Diagnostic accuracy of emergency-performed focused assessment with sonography for trauma (FAST) in blunt abdominal trauma.* Electronic Physician [Electronic Resource], 2016. **8**(9): p. 2950-2953. | Population |
| Ghane, M.R., et al., *Accuracy of early rapid ultrasound in shock (RUSH) examination performed by emergency physician for diagnosis of shock etiology in critically ill patients.* Journal of Emergencies Trauma & Shock, 2015. **8**(1): p. 5-10. | Population |
| Gratton, R., et al., *Diagnostic performance of the cardiac FAST in a high-volume Australian trauma centre.* Journal of Emergency Medicine, Trauma and Acute Care, 2017. **2017**(1). | Study type |
| Gul, W., A. Anjum, and A. Khan, *Diagnostic accuracy of abdominal ultrasonography in detection of hollow visceral injury in cases of blunt trauma abdomen.* Pakistan Journal of Medical and Health Sciences, 2018. **12**(1): p. 63-65. | Population |
| Hamada, S.R., et al., *Integrating eFAST in the initial management of stable trauma patients: the end of plain film radiography.* Annals of Intensive Care, 2016. **6**(1): p. 62. | Population |
| Hamid, S., N. Rasheed, and F. Rani, *Diagnostic accuracy of ultrasound in detection of visceral injury in blunt abdominal trauma.* Pakistan Journal of Medical and Health Sciences, 2014. **8**(4): p. 959-963. | Population |
| He, N.X., et al., *Clinical value of bedside abdominal sonography performed by certified sonographer in emergency evaluation of blunt abdominal trauma.* Chinese Journal of Traumatology, 2020. **23**(5): p. 280-283. | Population |
| Helmy, S., et al., *Role of chest ultrasonography in the diagnosis of lung contusion.* Egyptian Journal of Chest Diseases and Tuberculosis, 2015. **64**(2): p. 469-475. | Population |
| Heydari, F., M. Esmailian, and M. Dehghanniri, *Diagnostic Accuracy of Ultrasonography in the Initial Evaluation of Patients with Penetrating Chest Trauma.* Emergency (Tehran, Iran), 2014. **2**(2): p. 81-4. | Population |
| Huang, H.C., et al., *Lodox/Statscan facilitates the early detection of commonly overlooked extracranial injuries in patients with traumatic brain injury.* European Journal of Trauma & Emergency Surgery, 2012. **38**(3): p. 319-26. | Intervention |
| Huber-Wagner, S., et al., *Effect of the localisation of the CT scanner during trauma resuscitation on survival -- a retrospective, multicentre study.* Injury, 2014. **45**: p. S76-82. | In previous CPG version |
| Inaba, K., et al., *Cervical spinal clearance: A prospective Western Trauma Association Multi-institutional Trial.* The Journal of Trauma and Acute Care Surgery, 2016. **81**(6): p. 1122-1130. | Population |
| Iqbal, Y., et al., *Validity of the fast scan for diagnosis of intraabdominal injury in blunt abdominal trauma.* Journal of Ayub Medical College, Abbottabad: JAMC, 2014. **26**(1): p. 52-6. | Population |
| Jahanshir, A., et al., *Value of point-of-care ultrasonography compared with computed tomography scan in detecting potential life-threatening conditions in blunt chest trauma patients.* The Ultrasound Journal, 2020. **12**(1): p. 36. | Population |
| Jat, M.A., O.A. Bhattı, and A.R. Shoro, *Comparative assessment between clinical and CT scan diagnosis in the treatment of blunt abdominal trauma.* Annals of Clinical and Analytical Medicine, 2020. **11**: p. 243-247. | Population |
| Jiang, L., et al., *Comparison of whole-body computed tomography vs selective radiological imaging on outcomes in major trauma patients: a meta-analysis.* Scandinavian Journal of Trauma, Resuscitation & Emergency Medicine, 2014. **22**: p. 54. | Study type |
| Kalsoom, F., A. Rahim, and M. Saleem, *Diagnostic accuracy of ultrasonography in detection of gastrointestinal perforation among patients with blunt abdominal trauma.* Pakistan Journal of Medical and Health Sciences, 2016. **10**(4): p. 1312-1315. | Population |
| Karki, O.B., *The role of computed tomography in blunt abdominal trauma.* Journal of the Nepal Medical Association, 2015. **53**(200): p. 227-230. | Population |
| Kaya, S., et al., *A study on the evaluation of pneumothorax by imaging methods in patients presenting to the emergency department for blunt thoracic trauma.* Ulusal Travma ve Acil Cerrahi Dergisi = Turkish Journal of Trauma & Emergency Surgery: TJTES, 2015. **21**(5): p. 366-72. | Population |
| Khan, M.M., et al., *Frequency of abdominal ultrasonographics correct findings as compared with CT scan in detection of solid intra-abdominal visceral injuries.* Pakistan Journal of Medical and Health Sciences, 2014. **8**(2): p. 317-320. | Population |
| Kumar, S., et al., *Accuracy of Focused Assessment with Sonography for Trauma (FAST) in Blunt Trauma Abdomen-A Prospective Study.* Indian Journal of Surgery, 2015. **77**: p. 393-7. | Population |
| Kutcher, M.E., et al., *The role of computed tomographic scan in ongoing triage of operative hepatic trauma: A Western Trauma Association multicenter retrospective study.* The Journal of Trauma and Acute Care Surgery, 2015. **79**(6): p. 951-6; discussion 956. | Intervention |
| Langdorf, M.I., et al., *Yield and clinical predictors of thoracic spine injury from chest computed tomography for blunt trauma.* The Western Journal of Emergency Medicine, 2014. **15**(4): p. 465-70. | Population |
| Lee, C., et al., *Accuracy of Focused Assessment with Sonography for Trauma (FAST) in Disaster Settings: A Meta-Analysis and Systematic Review.* Disaster Medicine & Public Health Preparedness, 2019. **13**(5): p. 1059-1064. | Study type |
| Leichtle, S., et al., *Decreasing Accuracy of the eFAST Examination-Another Challenge Due to Morbid Obesity.* American Surgeon, 2019. **85**(8): p. 923-926. | Intervention |
| Mistral, T., et al., *Clinical Judgment Is Not Reliable for Reducing Whole-body Computed Tomography Scanning after Isolated High-energy Blunt Trauma.* Anesthesiology, 2017. **126**(6): p. 1116-1124. | Population |
| Montazer, S., et al., *Accuracy of Focused Assessment with Sonography for Trauma in Blunt Abdominal Trauma in Emergency Department.* Journal of Mazandaran University of Medical Sciences, 2016. **26**(140): p. 187-191. | Language |
| Moussavi, N., et al., *Routine versus selective chest and abdominopelvic CT-scan in conscious blunt trauma patients: a randomized controlled study.* European Journal of Trauma & Emergency Surgery, 2018. **44**(1): p. 9-14. | Population |
| Mumtaz, U., et al., *Bedside Ultrasonography: AUseful Tool for Traumatic Pneumothorax.* Jcpsp, Journal of the College of Physicians & Surgeons - Pakistan, 2016. **26**(6): p. 459-62. | Population |
| Mumtaz, U., et al., *Ultrasound And Supine Chest Radiograph In Road Traffic Accident Patients: A Reliable And Convenient Way To Diagnose Pleural Effusion.* Journal of Ayub Medical College, Abbottabad: JAMC, 2017. **29**(4): p. 587-590. | Population |
| Mushtaq, S., et al., *Diagnostic accuracy of Focused Abdominal Sonography in Trauma (FAST) in patients with blunt abdominal trauma.* Pakistan Journal of Medical and Health Sciences, 2014. **8**(2): p. 299-301. | Population |
| Musiitwa, P.C., et al., *Emergency Ultrasound Predicting the Need for Therapeutic Laparotomy among Blunt Abdominal Trauma Patients in a Sub-Saharan African Hospital.* Emergency Medicine International Print, 2014. **2014**: p. 793437. | Population |
| Nasr-Esfahani, M., M. Kolahdouzan, and M. Shafiei, *Ultrasound surface probe as a screening method for evaluating the patients with blunt abdominal trauma.* Journal of Research in Medical Sciences, 2014. **19**(1): p. 23-27. | Population |
| Netherton, S., et al., *Diagnostic accuracy of eFAST in the trauma patient: a systematic review and meta-analysis.* CJEM Canadian Journal of Emergency Medical Care, 2019. **21**(6): p. 727-738. | Population |
| Rehfeldt, M., et al., *Point-of-Care Diagnostic Device for Traumatic Pneumothorax: Low Sensitivity of the Unblinded PneumoScan TM.* Emergency Medicine International Print, 2018. **2018**: p. 7307154. | Population |
| Rodriguez, R.M., et al., *Diagnostic yields, charges, and radiation dose of chest imaging in blunt trauma evaluations.* Academic Emergency Medicine, 2014. **21**(6): p. 644-50. | Population |
| Rodriguez, R.M., et al., *Pneumothorax and Hemothorax in the Era of Frequent Chest Computed Tomography for the Evaluation of Adult Patients With Blunt Trauma.* Annals of Emergency Medicine, 2019. **73**(1): p. 58-65. | Study type |
| Sabri, Y.Y., et al., *Evaluating the role of ultrasound in chest trauma: Common complications and computed tomography comparative evaluation.* Egyptian Journal of Radiology and Nuclear Medicine, 2018. **49**(4): p. 986-992. | Population |
| Sabzghabaei, A., et al., *Pan vs. selective computed tomography scans in management ofmultiple trauma patients; a brief report.* Emergency, 2017. **5**(1). | Population |
| Scharonow, M. and C. Weilbach, *Prehospital point-of-care emergency ultrasound: a cohort study.* Scandinavian Journal of Trauma, Resuscitation & Emergency Medicine, 2018. **26**(1): p. 49. | Population |
| Shojaee, M., et al., *Diagnosis of intraabdominal injury after blunt abdominal trauma by combination of ultrasound, urine analysis and shock index.* Journal of Mazandaran University of Medical Sciences, 2014. **24**: p. 273-277. | Language |
| Sonhaye, L., et al., *Intravenous Contrast Medium Administration for Computed Tomography Scan in Emergency: A Possible Cause of Contrast-Induced Nephropathy.* Radiology Research & Practice Print, 2015. **2015**: p. 805786. | Population |
| Soult, M.C., et al., *Can routine trauma bay chest x-ray be bypassed with an extended focused assessment with sonography for trauma examination?* American Surgeon, 2015. **81**(4): p. 336-40. | Population |
| Staub, L.J., et al., *Chest ultrasonography for the emergency diagnosis of traumatic pneumothorax and haemothorax: A systematic review and meta-analysis.* Injury, 2018. **49**(3): p. 457-466. | Population |
| Stengel, D., et al., *Emergency ultrasound-based algorithms for diagnosing blunt abdominal trauma.* Cochrane Database of Systematic Reviews, 2015(9): p. CD004446. | Population |
| Treskes, K., et al., *Indications for total-body computed tomography in blunt trauma patients: a systematic review.* European Journal of Trauma & Emergency Surgery, 2017. **43**(1): p. 35-42. | In previous CPG version |
| Tunuka, C.E., et al., *Emergency sonography AIDS diagnostic accuracy of torso injuries: a study in a resource limited setting.* Emergency Medicine International Print, 2014. **2014**: p. 978795. | Population |
| Vafaei, A., et al., *Diagnostic Accuracy of Ultrasonography and Radiography in Initial Evaluation of Chest Trauma Patients.* Emergency (Tehran, Iran), 2016. **4**(1): p. 29-33. | Population |
| Vargas, C.A., et al., *Extension of the thoracic spine sign as a diagnostic marker for thoracic trauma.* European Journal of Trauma & Emergency Surgery, 2020. **17**: p. 17. | Population |
| Wirth, S., et al., *European Society of Emergency Radiology: guideline on radiological polytrauma imaging and service (short version).* Insights Into Imaging, 2020. **11**(1): p. 135. | Study type |
| Yates, J.G. and D. Baylous, *Aeromedical Ultrasound: The Evaluation of Point-of-care Ultrasound During Helicopter Transport.* Air Medical Journal, 2017. **36**(3): p. 110-115. | Study type |
| Yu, L., et al., *Blunt Traumatic Aortic Injury in the Pan-scan Era.* Academic Emergency Medicine, 2020. **27**(4): p. 291-296. | Intervention |
| Zhang, Z., et al., *Diagnostic accuracy of contrast enhanced ultrasound in patients with blunt abdominal trauma presenting to the emergency department: a systematic review and meta-analysis.* Scientific Reports, 2017. **7**(1): p. 4446. | Study type |
| Huang, R., et al., *Is magnetic resonance imaging becoming the new computed tomography for cervical spine clearance? Trends in magnetic resonance imaging utilization at a Level I trauma center.* The Journal of Trauma and Acute Care Surgery, 2020. **89**(2): p. 365-370 | Population |
| Mi, J., et al., *Prediction of MRI findings including disc injury and posterior ligamentous complex injury in neurologically intact thoracolumbar burst fractures by the parameters of vertebral body damage on CT scan.* Injury, 2018. **49**(2): p. 272-278 | Population |
| Ojaghi Haghighi, SH., et al., *Diagnostic value of bedside ultrasound for detecting cervical spine injuries in patients with severe multiple trauma.* Trauma Monthly, 2019. **24**(5) | No matching PICO |
| Resnick, S., et al., *Clinical relevance of magnetic resonance imaging in cervical spine clearance: a prospective study.* JAMA Surgery, 2014. **149**(9): p. 934-9 | Population |
| Songur Kodik, M., et al., *Computed tomography vs. magnetic resonance imaging in unstable cervical spine injuries.* Turkish Journal of Trauma & Emergency Surgery: TJTES, 2020. **26**(3): p. 431-438 | Study type |
| McCutcheon, L., et al., *Best practice in diagnostic imaging after blunt force trauma injury to the cervical spine: A systematic review.* Journal of Medical Imaging and Radiation Sciences, 2015. **46**(2): p. 231-240 | In previous CPG version |
| Efinger, K; Kildal, D.: Polytrauma: Thieme-Verlag | Study type |
| Chidambaram, S., E.L. Goh, and M.A. Khan, A meta-analysis of the efficacy of whole-body computed tomography imaging in the management of trauma and injury. Injury, 2017. 48(8): p. 1784-1793, 0020-1383, doi: 10.1016/j.injury.2017.06.003. | In previous CPG version |
| Pothmann C.E.M. et al.: Abdominalverletzungen des polytraumatisierten Erwachsenen – systematic review, Unfallchirurg (121), 2018, 159 – 173 | Study type |
| Müller AO-Frakturklassifikation für lange Röhrenknochen. AO-Foundation 2010 | Study type |
| Bühren V, Keel M, Marzi I, Augat P, Baas N, Beickert R, Botzlar A, Bräun K, Carrel T et al. Klassifikation des Weichteilschadens bei Frakturen. In: Bühren V, Keel M, Marzi I: Checkliste Traumatologie. 7. komplett überarbeitete und erweiterte Auflage. Stuttgart: Thieme; 2011. doi:10.1055/b-002-23575 | Study type |
| Kandziorra F et al.: Die AO-Spine-Klassifikation thorakolumbaler Wirbelsäulenverletzungen Z Orthop Unfall 2016; 154: 35–42 | Study type |
| Eurin, M., N. Haddad, M. Zappa, et al., Incidence and predictors of missed injuries in trauma patients in the initial hot report of whole-body CT scan. Injury, Int. Care Injured, 2012. 43(1): p. 73-77, 0020-1383, doi: https://doi.org/10.1016/j.injury.2011.05.019 | No matching PICO |
| Banaste, N., B. Caurier, F. Bratan, et al., Whole-Body CT in Patients with Multiple Traumas: Factors Leading to Missed Injury. Radiology, 2018. 289(2): p. 374-383, 0033-8419, doi: 10.1148/radiol.2018180492. | No matching PICO |
| Viergutz, T., T. Terboven, T. Henzler, et al., Relevante Zufallsbefunde und iatrogene Verletzungen : Eine retrospektive Analyse von 1165 Schockraumpatienten. Anaesthesist, 2018, 1432-055X, doi: 10.1007/s00101-018- 0505-7.) | No matching PICO |
| Treskes, K., S.A. Bos, L.F.M. Beenen, et al., High rates of clinically relevant incidental findings by total-body CT scanning in trauma patients; results of the REACT-2 trial. Eur Radiol, 2017. 27(6): p. 2451-2462, 1432-1084, doi: 10.1007/s00330-016-4598-6. | No matching PICO |
| Schicho, A., L. Luerken, R. Meier, et al., Incidence of traumatic carotid and vertebral artery dissections: results of cervical vessel computed tomography angiogram as a mandatory scan component in severely injured patients. Therapeutics and Clinical Risk Management, 2018. 14: p. 173-178, 1176-6336, doi: 10.2147/tcrm.S148176. | Study type |
| Fakler, J.K.M., O. Özkurtul, and C. Josten, Retrospective analysis of incidental non-trauma associated findings in severely injured patients identified by whole-body spiral CT scans. Patient Safety in Surgery, 2014. 8(36): p. 1-8, 1754-9493, doi: 10.1186/s13037-014-0036-3. | Study type |
| Clarke et al: Trauma 2002 March; 52 (3); 420 ff. | Intervention |
| ESER Guideline: Polytrauma – Langversion: s. S. 46; F.4.1 und F.4.2 | Study type |
| Hallinan, J.T.P.D., C.H. Tan, and U. Pua, The role of multidetector computed tomography versus digital subtraction angiography in triaging care and management in abdominopelvic trauma. Singapore Med J, 2016. 57(9): p. 497-502, doi: 10.11622/smedj.2015179. | No matching PICO |
| Colip, C.G., V. Gorantla, C.A. LeBedis, et al., Extremity CTA for penetrating trauma: 10-year experience using a 64-detector row CT scanner. Emerg Radiol, 2017. 24(3): p. 223-232, 1070-3004, doi: 10.1007/s10140-016-1469-z. | No matching PICO |
| Antoni, A, Heinz, T, Leitgeb, J: Polytrauma und begleitendes Schädel-Hirn-Trauma; Unfallchirurg 2017 (120) 722– 727 DOI - 10.1007/s00113- 017- 0354-x. | Study type |
| Hoffstetter P. et al.: Gibt es Notfallindikationen für die MRT? Notfall Rettungsmed 2012 · 15:516–520 | Study type |
| Braunschweig, R; Kaden, I: Polytrauma und Begutachtung in der Neurologie; Trauma und Berufskrankheit (7), 2005, 218 – 224 | Study type |
| Linsenmaier, U., L.L. Geyer, M. Korner, et al., Stellenwert der Multidetektor-CT bei Polytrauma. Radiologe, 2014. 54(9): p. 861-871, 0033-832x, doi: 10.1007/s00117-013-2634-y. | Intervention |
| Ernstberger: Computertomographie bei Polytrauma; TraumaBerufskrankh 2017 [1] 19: S57–S63 / DOI 10.1007/s10039-016-0204-z) | Intervention |
| Körner, M., L.L. Geyer, S. Wirth, et al., 64-MDCT in Mass Casualty Incidents: Volume Image Reading Boosts Radiological Workflow. AJR, 2011. 197(3): p. 399-404, 0361-803X, doi: 10.2214/AJR.10.5716. | Population |
| Leung, V., A. Sastry, T.D. Woo, et al., Implementation of a split-bolus single-pass CT protocol at a UK major trauma centre to reduce excess radiation dose in trauma pan-CT. Clinical Radiology, 2015. 70(10): p. 1110-1115, 0009-9260, doi: 10.1016/j.crad.2015.05.014. | Population |
| Hakim, W., R. Kamanahalli, E. Dick, et al., Trauma whole-body MDCT: an assessment of image quality in conventional dual-phase and modified biphasic injection. Br J Radiol, 2016. 89(20160160), 0007-1285, doi: 10.1259/bjr.20160160. | Population |
| Geyer, L.L., M. Körner, A. Harrieder, et al., Dose reduction in 64-row whole-body CT in multiple trauma: An optimized CT protocol with iterative image reconstruction on a gemstone-based scintillator. Br J Radiol, 2016. 89(20160003): p. 1-6, 0007-1285, doi: http://dx.doi.org/10.1259/bjr.20160003. | Population |
| Kahn, J., U. Grupp, D. Kaul, et al., Computed tomography in trauma patients using itera-tive reconstruction: reducing radiation exposure without loss of image quality. Acta Radiologica, 2016. 57(3): p. 362-369, 0284-1851, doi: 10.1177/0284185115580839. | No matching PICO |
| Fellner, F.A., J. Krieger, N. Lechner, et al., Polytrauma-Computertomographie : Technische Grundlagen, Workflow und Dosisreduktion. Radiologe, 2014. 54(9): p. 872-879, 0033- 832x, doi: 10.1007/s00117-013-2635-x. | Intervention |
| Kinoshita, T., K. Yamakawa, H. Matsuda, et al., The Survival Benefit of a Novel Trauma Workflow that Includes Immediate Whole-body Computed Tomography, Surgery, and Interventional Radiology, All in One Trauma Resuscitation Room: A Retrospective Historical Control Study. Ann Surg, 2017.XX(XX):p.1-7,doi: 10.1097/sla.0000000000002527. | Study type |
| Huber- Wagner, S., R. Lefering, L.M. Qvick, et al., Effect of whole-body CT during trauma resuscitation on survival: a retrospective, multicentre study. Lancet, 2009. 373(9673): p. 1455-1461, 0140-6736, doi: 10.1016/s0140-6736(09)60232-4. | In previous CPG version |
| Davies, R.M., A.B. Scrimshire, L. Sweetman, et al., A decision tool for whole-body CT in major trauma that safely reduces unnecessary scanning and associated radiation risks: An initial exploratory analysis. Injury, Int. Care Injured, 2016. 47(1): p. 43-49, 0020-1383, doi: 10.1016/j.injury.2015.08.036. | Intervention |
| QS-LL-CT: Polytrauma der BÄK – erscheint in 2022 | Study type |
| Wirth S. and Hebebrand J., Basilico R., Berger F. H., et al., European Society of Emergency Radiology - Guideline on Radiological Polytrauma Imaging and Service (short version). Insights into Imaging, accepted: 09.11.2020 | Study type |
| Geyer, L.L., M. Körner, U. Linsenmaier, et al., Incidence of delayed and missed diagnoses in whole-body multidetector CT in patients with multiple injuries after trauma. Acta Radiologica, 2013. 54(5): p. 592-598, 0284-1851, doi: 10.1177/0284185113475443 | No matching PICO |
| Reske, S.U., R. Braunschweig, A.W. Reske, et al., Whole-Body CT in Multiple Trauma Patients: Clinically Adapted Usage of Differently Weighted CT Protocols. Fortschr Röntgenstr, 2018. 190(12): p. 1141-1151, 1438-9029, doi: https://doi.org/10.1055/a-0643-4553. | Population |
| Frellesen, C., M. Boettcher, J.L. Wichmann, et al., Evaluation of a dual-room sliding gantry CT concept for workflow optimisation in polytrauma and regular in- and outpatient management. European Journal Radiology, 2015. 84(1): p. 117-122, 0720-048x, doi: 10.1016/j.ejrad.2014.10.013. | Population |
| Huber-Wagner, S., C. Mand, S. Ruchholtz, et al., Effect of the localisation of the CT scanner during trauma resuscitation on survival—A retrospective, multicentre study. Injury, Int. Care Injured, 2014. 45: p. 76-82, 0020-1383, doi: https://doi.org/10.1016/j.injury.2014.08.022 | In previous CPG version |
| Jöres, A.P., J.T. Heverhagen, H. Bonel, et al., Diagnostic Accuracy of Full-Body Linear X-Ray Scanning in Multiple Trauma Patients in Comparison to Computed Tomography. Fortschr Röntgenstr, 2016. 188(2): p. 163-171, 1438-9010, doi: 10.1055/s-0041-107199 | Study type |
| British Orthopaedic Association, Association of Surgeons of Great Britain and Ireland, and The Association of Coloproctology of Great Britain and Ireland. THE MANAGEMENT OF PATIENTS WITH PELVIC FRACTURES. 2018 | Population |
| The Royal College of Radiologists. Standards of practice and guidance for trauma radiology in severely injured patients. 2015 London: The Royal College of Radiologists, Second Edition, Available from: https://www.rcr.ac.uk/system/files/publication/field_publication_files/bfcr155_traumaradiol.pdf. Accessed 29.01. 2019 | Study type |
| [National Institute for Health and Care Excellence, Head injury: assessment and early management: guidance (cg176). 2014, ISBN: 978-1-4731-0405-1, Available from: https://www.nice.org.uk/guidance/cg176.](http://?) | Study type |
| Summerton, D.J., N. Djakovic, N.D. Kitrey, et al. Guidelines on Urological Trauma. European Association of Urology (EAU) 2014, Update: 2015 Available from: http://uroweb.org/wp-content/uploads/EAU-Guidelines-Urological-Trauma_LRV2.pdf. Accessed 16.01. 2019 | Study type |
| Mück, F., K. Wirth, M. Muggenthaler, et al., Prätherapeutische Ablaufanalyse bei einem Massenanfall von Verletzten: Vergleich von zwei Traumazentren der höchsten Versorgungsstufe. Unfallchirurg, 2016. 119(8): p. 632-641, 0177-5537, doi: 10.1007/s00113-016-0200-6). | Study type |
| Zieleskiewicz, L., R. Fresco, G. Duclos, et al., Integrating extended focused assessment with sonography for trauma (eFAST) in the initial assessment of severe trauma: Impact on the management of 756 patients. Injury, Int. J. Care Injured, 2018. 49(10): p. 1774-1780, 0020-1383, doi: 10.1016/j.injury.2018.07.002. | Study type |
| Deutsche Gesellschaft für Verbrennungsmedizin (DGV), R.u.Ä.C.D. Deutsche Gesellschaft der Plastischen, Deutsche Interdisziplinäre Vereinigung für Anästhesiologie und Intensivmedizin e. V. (DIVI), et al. S2k - Behandlung thermischer Verletzungen des Erwachsenen (AWMF-Registernr.: 044-001). 2018, Available from: https://www.awmf.org/leitlinien/detail/ll/044-001.html. Accessed 04.12. 2018), | Study type |
| Deutsche Gesellschaft für Gastroenterologie Verdauungs- und Stoffwechselkrankheiten (DGVS), Deutsche Röntgengesellschaft (DRG), Deutsche Gesellschaft für Allgemein- und Viszeralchirurgie e.V. (DGAV), et al. S2k Leitlinie Gastrointestinale Blutung (AWMF-Registernr.: 021/028). 2017, Available from: https://www.awmf.org/uploads/tx_szleitlinien/021-028l_S2k_Gastrointestinale_Blutung_2017-07.pdf. Accessed 29.01. 2019 | Study type |
| National Institute for Health and Care Excellence, Major trauma: assessment and initial management: guidance (ng39). 2016, ISBN: 978-1-4731-1680-1, Available from: https://www.nice.org.uk/guidance/ng39. | Study type |
| Diercks, D.B., A. Mehrotra, D.J. Nazarian, et al., Clinical Policy: Critical Issues in the Evaluation of Adult Patients Presenting to the Emergency Department With Acute Blunt Abdominal Trauma. Ann Emerg Med, 2011. 57(4): p. 387-404, 0196-0644, doi: 10.1016/j.annemergmed.2011.01.013. | Study type |
| Stengel, D., G. Rademacher, A. Ekkernkamp, et al., Emergency ultrasound-based algorithms for diagnosing blunt abdominal trauma (Review). Cochrane Database Systematic Reviews, 2015(9): p. 1-38, 1361-6137, doi: 10.1002/14651858.CD004446.pub4. | Study type |
| Schieb E et al.: Notfallsonographie. Anaesthesist 2015 · 64:329–344 | Study type |
| Goldstein, S.A., A. Evangelista, S. Abbara, et al., Multimodality Imaging of Diseases of the Thoracic Aorta in Adults: From the American Society of Echocardiography and the European Association of Cardiovascular Imaging : Endorsed by the Society of Cardiovascular Computed Tomography and Society for Cardiovascular Magnetic Resonance. J Am Soc Echocardiogr, 2015. 28(2): p. 119-182, 0894-7317, doi: http://dx.doi.org/10.1016/j.echo.2014.11.015.) | Study type |
| Sauter, T.C., S. Hoess, B. Lehmann, et al., Detection of pneumothoraces in patients with multiple blunt trauma: use and limitations of eFAST. Emerg Med J, 2017. 34(9): p. 568-572, 1472-0205, doi: 10.1136/emermed-2016-205980. | Study type |
| Huber-Wagner, S., R. Lefering, L.M. Qvick, et al., Effect of whole-body CT during trauma resuscitation on survival: a retrospective, multicentre study. Lancet, 2009. 373(9673): p. 1455-1461, 0140-6736, doi: 10.1016/s0140-6736(09)60232-4. | In previous CPG version |
| Alagic, Z., A. Eriksson, E. Drageryd, et al., A new low-dose multi-phase trauma CT protocol and its impact on diagnostic assessment and radiation dose in multi-trauma patients. Emerg Radiol, 2017. 24(5): p. 509-518, 1438-1435, doi: 10.10007/s10140-017-1496-4. | Population |
| Colip, C.G., V. Gorantla, C.A. LeBedis, et al., Extremity CTA for penetrating trauma: 10-year experience using a 64-detector row CT scanner. Emerg Radiol, 2017. 24(3): p. 223-232, 1070-3004, doi: 10.1007/s10140-016-1469-z. | No matching PICO |
| Hsiao, K.H., M.M. Dinh, K.P. McNamara, et al., Whole-body computed tomography in the initial assessment of trauma patients: Is there optimal criteria for patient selection? Emergency Medicine Australasta, 2013. 25(2): p. 182-191, 1742-6723, doi: 10.1111/1742-6723.12041 | Population |
| Saltzherr, T.P., F.C. Bakker, L.F. Beenen, et al., Randomized clinical trial comparing the effect of computed tomography in the trauma room versus the radiology department on injury outcomes. British Journal of Surgery, 2012. 99(1): p. 105‐113, doi: 10.1002/bjs.7705 | In previous CPG version |

# S4 Evidence Table

##### FAST for the diagnosis of free fluid after blunt or penetrating abdominal trauma.

| Study: Reference, aim, design, setting | Participants: selection criteria, characteristics | N Participants;  Intervention (IG) vs. Control group (CG) | Main outcomes | Assessment: LoE, risk of bias; Conclusions |
| --- | --- | --- | --- | --- |
| Akdemir (2019)  "The Blunt Abdominal Trauma Bedside Ultrasonography Comparison with Trauma Severity Scores and Computerized Tomography ". *Jcpsp, Journal of the College of Physicians & Surgeons – Pakistan* 2019; 29(7): 621-625  Study design  Diagnostic cross-sectional study  Aim of the study  “The aim of the present study was to investigate the relationship between the application of FAST performed by emergency physicians in patients with blunt trauma and the management, clinical outcome, prognosis, and trauma severity scores for such patients.”  Setting  Turkey, 2013-2017 | Inclusion criteria   - Patients admitted to the emergency department (ED) because of blunt trauma - aged 18 years or older   Exclusion criteria   - children <18 years with blunt abdominal trauma - patients who did not want to be involved in the study for any reason - patients who were admitted in the absence of trained personnel for FAST application - patients diagnosed by CT only and without FAST   Characteristics  Age [y], mean (± SD)  37.7 (±17.3)  Male, n (%)  219 (69.5)  FAST - presence of free fluid, n (%)  28 (8.9)  Patients undergoing surgery, n (%)  95 (30.1) | Participants  N=315 patients admitted to the ED with blunt abdominal trauma  Tests evaluated  Index test: FAST, ultrasound investigation to detect free fluid (FF), done by experienced emergency physicians (emergency medicine specialists and/or emergency medicine assistants)  Reference standard: Intravenous contrast-enhanced CT (gold standard) was used to assess the presence of intraperitoneal FF in patients with indications to obtain CT imaging by radiologists | Primary outcomes  Mortality, n (%)  FAST: 11 (3.5)  Diagnostic accuracy for detection of free fluid (FAST)  True positive, N=28  False positive, N=0  True negative, N=134  False negative, N=6  Sensitivity, % (95% CI)^§^  82.3 (65.4-93.2)  Specificity, % (95% CI)^§^  100 (97.2-100)  PPV, % (95% CI)^§^  100 (91.5-97.8)  NPV, % (95% CI)^§^  95.7 (91.5-97.8)  ^§^compared to CT as gold standard  FAST is strongly compatible with CT (κ= 0.882, p<0.001) | Level of evidence  2b  Risk of bias (QUADAS)  Patient selection: +  Index test: ?  Reference standard: ?  Flow and timing: ?  Authors’ conclusion  “Early and appropriate FAST practice provides valuable and prognostic information. FAST prevents time delays and transportation out of the emergency department in the evaluation of hemodynamically unstable patients.”  Reviewers’ conclusion  There are unclear risk of bias concerning the index test, reference standard and flow and timing as necessary information were not provided in the article.  The results of the study should be interpreted with caution. Furthermore, the authors conclude that FAST prevents time delays in hemodynamically instable patients, but times were not reported and cannot be considered as relevant in this context. |
| Akoglu (2017)  “Diagnostic accuracy of the Extended Focused Abdominal Sonography for Trauma (E-FAST) performed by emergency physicians compared to CT”. *American Journal of Emergency Medicine* 2018; 36 (6): 1014-1017  Study design  Diagnostic cross-sectional study  Aim of the study  „The aim of this study was to compare the diagnostic accuracy of the E-FAST exam performed by emergency medicine (EM) residents with the results of CT scan as a gold standard.”  Setting  US, 2014-2015 | Inclusion criteria   - Patients ≥18 years - Multiple trauma (defined according to ATLS as more than one anatomical area was affected) - Any patient in whom thoraco-abdominal CT was ordered during the shifts where the researchers were on duty   Exclusion criteria   - Unstable patients (systolic blood pressure (SBP) b 100 mm Hg and/or heart rate (HR) N 100 beats/min and/or ≥4 U of packed red blood cells transfused in the trauma bay - Patients unavailable for CT (unable to leave the trauma bay for CT, died in the ED, referred to the operating room before CT) - Pregnant patients - Patients intubated - Patients with anatomical defect(s) at the site of sonographic imaging, - Patients with known allergies to contrast materials   Characteristics  Age [y], median (IQR)  38 (30-49)  Male, n (%)  102 (79.1)  SBP (mm Hg), mean (SD), (95% CI)  127.9 (20.5), (124.3, 131.5) | Participants  N=144 trauma patients  Finally: 132 for abdominal, 130 for thorax examinations analysed  Tests evaluated  Index test: e-FAST; all sonographic examinations were performed by two senior EM residents (OFC, AC) who were certified for bedside sonography with 3 years of experience in protocols such as E-FAST, RUSH, or POCUS with an average of 500 bedside US examinations per year. When a trauma patient was ordered to have a thoraco-abdominal CT by the attending EP, an E-FAST examination was performed before the patient has left the ED for CT, if not already. E-FAST exam findings were recorded on a study chart before the CT examination.  Reference standard: computed tomography scans; CTs were evaluated by a radiology specialist who was blinded to the patients and results of the sonographic examinations. A senior academic radiology faculty (RE) who was blinded to patients, and sonographic findings reviewed the images and official radiology reports, and her reports were used as the gold standard. A dedicated MDCT was used for all ED imaging which had PACS capabilities (Picture Archiving and Communication System) and all examinations were contrast-enhanced according to local trauma protocol. | Primary outcomes  Diagnostic accuracy for detection of abdominal free fluid (E-FAST)  True positive, N=3  False positive, N=2  True negative, N=123  False negative, N=4  Sensitivity, % (95% CI)  42.9 (9.9, 81.6)  Specificity, % (95% CI)  98.4 (94.3, 99.8)  AUC (95% CI)  0.71 (0.62, 0.78)  +LR (95% CI)  26.8 (5.3, 135,2)  –LR (95% CI)  0.58 (0.31, 1.1)  *Cave: other outcomes concerning diagnostic accuracy for detection of pneumothorax are reported under 2.123* | Level of evidence  2b  Risk of bias (QUADAS)  Patient selection: ?  Index test: +  Reference standard: +  Flow and timing: +  Authors’ conclusion  “E-FAST examination has an excellent specificity. However, the sensitivity of the test is not high enough to rule-out thoraco-abdominal injuries in trauma patients when performed by EM residents.”  Reviewers’ conclusion  There is an unclear risk of selection bias in this study as patients were included in the study when decision for CT was already made during assessment because of clinical suspicion for thoraco-abdominal injuries. |
| Bagheri-Hariri (2019)  „The effect of extended-focused assessment with sonography in trauma results on clinical judgment accuracy of the physicians managing patients with blunt thoracoabdominal trauma”. *Archives of Trauma Research* 2019; 8(4): 207-213  Study design  Diagnostic cross-sectional study  Aim of the study  “Therefore, we decided to examine the effect of using E‑FAST in the clinical judgment of the physicians managing patients with blunt abdominal and chest wall trauma.”  Setting  Iran, 2014-2015 | Inclusion criteria   - Patients who were admitted to the ED with an abdominal or chest blunt trauma and for whom E‑FAST was conducted   Exclusion criteria   - Patients with penetrating trauma   Characteristics (participants)  Age [y], mean (±SD)  36.2 (±12.37)  Male, n (%)  90 (80) | Participants  N=115 patients with blunt abdominal or chest trauma  Tests evaluated  Index Test 1: Ph/E, Physical examination  Index Test 2: Ph/E + E-FAST, physical examination and additional extended‑focused assessment with sonography in trauma  Reference standard: findings in CT examination or intraoperative findings  Study interventions   - On arrival to the ED, based on the Advanced Trauma Life Support (ATLS) Guideline, the patients had undergone a primary resuscitation, and airway status, head and neck condition, and vital signs status including blood pressure, heart rate, respiratory rate, and Glasgow coma score were investigated. - For data collection a checklist with three parts was used. - Part I: Patient’s basic information was collected in a checklist and the revised trauma score (RTS) was calculated - Part II: Possible consequences based on the primary clinical judgment, the results from E‑FAST on existence or nonexistence of free fluid, and possible consequences according to the results obtained from the E‑FAST were recorded - Part III: Actual outcome of patient’s condition in the first 24 h (patient discharge without a follow‑up order, patient discharge with a follow‑up order, patient admission in general ward and/or intensive care unit, surgical intervention, and patient’s death) and also patient’s condition during the first 28 days - The prediction power of E‑FAST in traumatic patients was assessed. | Primary outcomes  Diagnostic accuracy for detection of haemorrhagic shock (Ph/E)^1^  True positive, N=13  False positive, N=2  True negative, N=98  False negative, N=2  Sensitivity, % (95% CI)  86.7 (59.5-98.3)  Specificity, % (95% CI)  98.0 (93.0-99.8)  PPV, % (95% CI)  86.7 (59.5-98.3)  NPV, % (95% CI)  98.0 (93.0-99.8)  AUC (95% CI)  0.92 (0.86-0.96)  Diagnostic accuracy for detection of haemorrhagic shock (Ph/E + E-FAST)^1^  True positive, N=12  False positive, N=2  True negative, N=98  False negative, N=3  Sensitivity, % (95% CI)  80.0 (51.9-95.7)  Specificity, % (95% CI)  98.0 (93.0-99.8)  PPV, % (95% CI)  85.7 (57.2-98.2)  NPV, % (95% CI)  97.0 (91.6-99.4)  AUC (95% CI)  0.89 (0.82-0.94)  ^1^Ƙ= 0.803  Diagnostic accuracy for detection of hemoperitoneum (Ph/E)^3^  True positive, N=5  False positive, N=2  True negative, N=100  False negative, N=8  Sensitivity, % (95% CI)  38.5 (13.9-68.4)  Specificity, % (95% CI)  98.0 (93.1-99.8)  PPV, % (95% CI)  71.4 (29.0-96.3)  NPV, % (95% CI)  92.6 (85.9-96.7)  AUC (95% CI)  0.68 (0.59-0.77)  Diagnostic accuracy for detection of hemoperitoneum (Ph/E + E-FAST)^3^  True positive, N=10  False positive, N=0  True negative, N=102  False negative, N=3  Sensitivity, % (95% CI)  76.9 (46.2-95.0)  Specificity, % (95% CI)  100 (96.5-100)  PPV, % (95% CI)  100 (69.1-100)  NPV, % (95% CI)  97.1 (91.9-99.4)  AUC (95% CI)  0.88 (0.81-0.94)  ^3^Ƙ= 0.430  Diagnostic accuracy for detection of solid organ damage (Ph/E)^4^  True positive, N=3  False positive, N=3  True negative, N=101  False negative, N=8  Sensitivity, % (95% CI)  27.3 (6.0-61.0)  Specificity, % (95% CI)  97.1 (91.8-99.4)  PPV, % (95% CI)  50.0 (11.8-88.2)  NPV, % (95% CI)  92.7 (86.1-96.8)  AUC (95% CI)  0.62 (0.53-0.71)  Diagnostic accuracy for detection of solid organ damage (Ph/E + E-FAST)^4^  True positive, N=10  False positive, N=0  True negative, N=104  False negative, N=1  Sensitivity, % (95% CI)  90.9 (58.7-99.8)  Specificity, % (95% CI)  100 (96.5-100)  PPV, % (95% CI)  100 (69.2-100)  NPV, % (95% CI)  99.0 (94.8-100)  AUC (95% CI)  0.95 (0.90-0.98)  ^4^Ƙ= 0.331  The values of AUC revealed that except for hemorrhagic shock, E‑FAST significantly increases the accuracy of diagnosis of posttraumatic complications, including hemoperitoneum, solid organ damage, and pneumothorax and hemothorax.  Cave: outcomes for pneumothorax and hemothorax are reported under 2.123). | Level of evidence  2b  Risk of bias (QUADAS)  Patient selection: ?  Index test: +  Reference standard: ?  Flow and timing: ?  Authors’ conclusion  “The results of this study showed that performing an E‑FAST increases the sensitivity of history and physical examination in diagnosis of pneumothorax, hemoperitoneum, solid organ damage, and hemothorax. It can be reported that except for hemorrhagic shock, E‑FAST significantly increases the accuracy of diagnosis.”  Reviewers’ conclusion  There is an unclear risk of bias in patient selection as the study chose a convenient sampling. It is unclear whether examiners were blinded to the results of the index test. Furthermore, there is an unclear risk of bias according to the patient flow, as not all patients received the same reference standard test. |
| Zanobetti (2018)  „Chest Abdominal‑Focused Assessment Sonography for Trauma during the primary survey in the Emergency Department: the CA‑FAST protocol” *European Journal of Trauma Emergency Surgery* (2018); 44: 805-810  Study design  Diagnostic cross-sectional study  Aim of the study  „(…)we developed a new protocol (CA-FAST, Chest Abdominal FAST) that integrates the detection of LCs in the E-FAST examination which can be performed during the primary survey. The aim of this study was to evaluate the feasibility and the diagnostic performance of CA-FAST examination when compared to the gold standard, thoracoabdominal CT.”  Setting  Italy, 2012-2013 | Inclusion criteria   - Adult trauma patients presenting to the ED - Underwent a thoracoabdominal CT scan were enrolled if a CA-FAST examination was previously performed   Exclusion criteria  n.r.  Characteristics (participants)  Age [y], mean (±SD)  46 (±20)  Male, n (%)  467 (75)  ISS, mean (±SD)  16 (±12) | Participants  N=601 trauma patients  Tests evaluated  Index test: Chest Abdominal Focussed Assessment Sonography for Trauma (CA-FAST) protocol, CA-FAST protocol consisted of a combined ultrasonographic evaluation of chest and abdomen in order to detect the presence of the following ultrasonographic patterns: pneumothorax (PTX), pleural effusion (PE), lung contusion (LC), pericardial and intraperitoneal effusion. The full examination consisted of 8 chest scans and 4 abdominal scans acquired with the patient in obligated supine position due to spinal boards and cervical collars. Chest US was performed by a 4- to 8-MHz linear probe or a 3.5- to 5-MHz curved array probe. Performance of CA-FAST by 12 emergency physicians. The abdominal US was performed by 5-MHz curved array probe using the standard 4-views.  Reference standard: thoracoabdominal CT scan, scans were enrolled if a CA-FAST examination was previously performed; CT scan was either required or not at discretion of the emergency physician (EP), independently of patient’s participation to the study. | Primary outcomes  Diagnostic accuracy for detection of free fluid (abdominal 4-view FAST)  Sensitivity, % (95% CI)  75 (67–83)  Specificity, % (95% CI)  96 (93–97)  PPV, % (95% CI)  81 (73–88)  NPV, % (95% CI)  94 (91–96)  +LR, % (95% CI)  17 (11–26)  −LR, % (95% CI)  0.3 (0.2–0.4)  Accuracy, % (95% CI)  91 (85–93)  *Note: more relevant outcomes concerning pneumothorax are reported under 2.123.* | Level of evidence  3b  Risk of bias (QIADAS)  Patient selection: ?  Index test: +  Reference standard: +  Flow and timing:?  Authors’ conclusion  „In summary CA-FAST protocol, performed in the emergency setting, showed important advantages: It is a noninvasive, rapid, ionizing radiation-free and an easily repeatable method; in trauma patients it allows to accurately and immediately detect diagnostic information and ultrasonographic patterns of severe injury. Moreover, the addition of four chest scans and the research of LCs did not cause a delay in the diagnosis.(…) CA-FAST protocol could represent an integrative tool of traditional CT scan in the management of trauma patients; it should be used as the initial investigation, during the primary survey, sending to further diagnostic studies only patients not clearly classified.”  Reviewers’ conclusion  There is an unclear risk of patient selection bias in this study as no further information about exclusion criteria was provided. Missing information about time intervals between the examinations lead to the conclusion of unclear risk for bias in the flow and timing. |
| +: low risk; –: high risk; ?: unclear risk; CI: Confidence Interval; E-Fast: extended Focused Abdominal Sonography for Trauma; CT: computed tomography; IQR: Interquartile Range; OR: Odds Ratio; SD: Standard Deviation; PPV: Positive Predictive Value; NPV: Negative Predictive Value; AUC: Area under the curve; adj.: adjusted; d: days; m: months; y: years; min: minutes | | | | |

##### Diagnosis of pneumothorax or hematothorax by transthoracic ultrasonography, repeat sonographic examinations.

| Study: Reference, aim, design, setting | Participants: selection criteria, characteristics | N Participants;  Intervention (IG) vs. Control group (CG) | Main outcomes | Assessment: LoE, risk of bias; Conclusions |
| --- | --- | --- | --- | --- |
| Akoglu (2017)  *see 2.121*  “Diagnostic accuracy of the Extended Focused Abdominal Sonography for Trauma (E-FAST) performed by emergency physicians compared to CT”. *American Journal of Emergency Medicine* 2018; 36 (6): 1014-1017  Study design  Diagnostic cross-sectional study  Aim of the study  „The aim of this study was to compare the diagnostic accuracy of the E-FAST exam performed by emergency medicine (EM) residents with the results of CT scan as a gold standard.”  Setting  US, 2014-2015 | Inclusion criteria   - Patients ≥18 years - Multiple trauma (defined according to ATLS as more than one anatomical area was affected) - Any patient in whom thoraco-abdominal CT was ordered during the shifts where the researchers were on duty   Exclusion criteria   - Unstable patients (systolic blood pressure (SBP) b 100 mm Hg and/or heart rate (HR) N 100 beats/min and/or ≥4 U of packed red blood cells transfused in the trauma bay - Patients unavailable for CT (unable to leave the trauma bay for CT, died in the ED, referred to the operating room before CT) - Pregnant patients - Patients intubated - Patients with anatomical defect(s) at the site of sonographic imaging, - Patients with known allergies to contrast materials   Characteristics  Age [y], median (IQR)  38 (30-49)  Male, n (%)  102 (79.1)  SBP (mm Hg), mean (SD), (95% CI)  127.9 (20.5), (124.3, 131.5) | Participants  N=144 trauma patients  Finally: 132 for abdominal, 130 for thorax examinations analysed  Tests evaluated  Index test: e-FAST; all sonographic examinations were performed by two senior EM residents (OFC, AC) who were certified for bedside sonography with 3 years of experience in protocols such as E-FAST, RUSH, or POCUS with an average of 500 bedside US examinations per year. When a trauma patient was ordered to have a thoraco-abdominal CT by the attending EP, an E-FAST examination was performed before the patient has left the ED for CT, if not already. E-FAST exam findings were recorded on a study chart before the CT examination.  Reference standard: computed tomography scans; CTs were evaluated by a radiology specialist who was blinded to the patients and results of the sonographic examinations. A senior academic radiology faculty (RE) who was blinded to patients, and sonographic findings reviewed the images and official radiology reports, and her reports were used as the gold standard. A dedicated MDCT was used for all ED imaging which had PACS capabilities (Picture Archiving and Communication System) and all examinations were contrast-enhanced according to local trauma protocol. | Primary outcomes  Diagnostic accuracy for detection of pleural effusion (E-FAST)  True positive, N=2  False positive, N=0  True negative, N=128  False negative, N=0  Sensitivity, % (95% CI)  100.0 (15.8, 100.0)  Specificity, % (95% CI)  100.0 (97.2, 100.0)  AUC (95% CI)  1.0 (0.97, 1.0)  Diagnostic accuracy for detection of pneumothorax (E-FAST)  True positive, N=6  False positive, N=1  True negative, N=121  False negative, N=2  Sensitivity, % (95% CI)  75.0 (35.0, 96,8)  Specificity, % (95% CI)  99.2 (95.5, 100.0)  AUC (95% CI)  0.87 (0.80, 0.92)  Positive Likelihood Ratio (+LR) (95% CI)  91.5 (12.5, 671.1)  Negative Likelihood Ratio (-LR) (95% CI)  0.25 (0.08, 0.84)  *Note: other outcomes of this study are reported under 2.121* | Level of evidence  2b  Risk of bias (QUADAS)  Patient selection: ?  Index test: +  Reference standard: +  Flow and timing: +  Authors’ conclusion  “E-FAST examination has an excellent specificity. However, the sensitivity of the test is not high enough to rule-out thoraco-abdominal injuries in trauma patients when performed by EM residents.”  Reviewers’ conclusion  There is an unclear risk of selection bias in this study as patients were included in the study when decision for CT was already made during assessment because of clinical suspicion for thoraco-abdominal injuries. |
| Bagheri-Hariri (2019)  *see also 2.121*  „The effect of extended-focused assessment with sonography in trauma results on clinical judgment accuracy of the physicians managing patients with blunt thoracoabdominal trauma”. *Archives of Trauma Research* 2019; 8(4): 207-213  Study design  Diagnostic cross-sectional study  Aim of the study  “Therefore, we decided to examine the effect of using E‑FAST in the clinical judgment of the physicians managing patients with blunt abdominal and chest wall trauma.”  Setting  Iran, 2014-2015 | Inclusion criteria   - Patients who were admitted to the ED with an abdominal or chest blunt trauma and for whom E‑FAST was conducted   Exclusion criteria   - Patients with penetrating trauma   Characteristics (participants)  Age [y], mean (±SD)  36.2 (±12.37)  Male, n (%)  90 (80) | Participants  N=115 patients with blunt abdominal or chest trauma  Tests evaluated  Index Test 1: Ph/E, Physical examination  Index Test 2: Ph/E + E-FAST, physical examination and additional extended‑focused assessment with sonography in trauma  Reference standard: findings in CT examination or intraoperative findings  Study interventions   - On arrival to the ED, based on the Advanced Trauma Life Support (ATLS) Guideline, the patients had undergone a primary resuscitation, and airway status, head and neck condition, and vital signs status including blood pressure, heart rate, respiratory rate, and Glasgow coma score were investigated. - For data collection a checklist with three parts was used. - Part I: Patient’s basic information was collected in a checklist and the revised trauma score (RTS) was calculated - Part II: Possible consequences based on the primary clinical judgment, the results from E‑FAST on existence or nonexistence of free fluid, and possible consequences according to the results obtained from the E‑FAST were recorded - Part III: Actual outcome of patient’s condition in the first 24 h (patient discharge without a follow‑up order, patient discharge with a follow‑up order, patient admission in general ward and/or intensive care unit, surgical intervention, and patient’s death) and also patient’s condition during the first 28 days. - The prediction power of E‑FAST in traumatic patients was assessed. | Primary outcomes  Diagnostic accuracy for detection of pneumothorax (Ph/E)^2^  True positive, N=5  False positive, N=6  True negative, N=103  False negative, N=1  Sensitivity, % (95% CI)  83.3 (35.9-99.6)  Specificity, % (95% CI)  94.5 (88.4-97.9)  PPV, % (95% CI)  45.5 (16.7-76.6)  NPV, % (95% CI)  99.0 (94.8-100)  AUC (95% CI)  0.89 (0.82-0.94)  Diagnostic accuracy for detection of pneumothorax (Ph/E + E-FAST)^2^  True positive, N=10  False positive, N=2  True negative, N=102  False negative, N=1  Sensitivity, % (95% CI)  90.9 (58.7-99.8)  Specificity, % (95% CI)  98.1 (93.2-99.8)  PPV, % (95% CI)  83.3 (51.6-97.9)  NPV, % (95% CI)  99.0 (94.7-100)  AUC (95% CI)  0.94 (0.89-0.98)  ^2^Ƙ= 0.642  Diagnostic accuracy for detection of hemothorax (Ph/E)^5^  True positive, N=1  False positive, N=1  True negative, N=109  False negative, N=4  Sensitivity, % (95% CI)  20.0 (0.51-71.6)  Specificity, % (95% CI)  99.1 (95.0-100)  PPV, % (95% CI)  50.0 (1.3-98.7)  NPV, % (95% CI)  96.5 (91.2-99.0)  AUC (95% CI)  0.60 (0.50-0.69)  Diagnostic accuracy for detection of hemothorax (Ph/E + E-FAST)^5^  True positive, N=4  False positive, N=0  True negative, N=110  False negative, N=1  Sensitivity, % (95% CI)  80.0 (28.4-99.5)  Specificity, % (95% CI)  100 (96.7-100)  PPV, % (95% CI)  100 (39.8-100)  NPV, % (95% CI)  99.1 (95.1-100)  AUC (95% CI)  0.90 (0.83-0.95)  ^5^Ƙ= 0.318  Note: other outcomes of this study were reported under 2.121 | Level of evidence  2b  Risk of bias (QUADAS)  Patient selection: ?  Index test: +  Reference standard: ?  Flow and timing: ?  Authors’ conclusion  “The results of this study showed that performing an E‑FAST increases the sensitivity of history and physical examination in diagnosis of pneumothorax, hemoperitoneum, solid organ damage, and hemothorax. It can be reported that except for hemorrhagic shock, E‑FAST significantly increases the accuracy of diagnosis.”  Reviewers’ conclusion  There is an unclear risk of bias in patient selection as the study chose a convenient sampling. It is unclear whether examiners were blinded to the results of the index test. Furthermore, there is an unclear risk of bias according to the patient flow, as not all patients received the same reference standard test. |
| Ezzat (2018)  „Evaluation of the role of bedside ultrasonography in the detection of traumatic occult pneumothorax”. *Journal of the Egyptian Society of Cardio-Thoracic Surgery* 2018; 26(2): 146-150  Study design  Diagnostic cross-sectional study  Aim of the study  “The aim of the study was to evaluate the role of bedside thoracic ultrasonography (U/S) for detection of occult pneumothorax in patients with chest trauma.”  Setting  Egypt, 2016-2018 | Inclusion criteria   - Age ≥18 years - Polytraumatized patients with trauma experienced within the same day of presentation in the emergency department   Exclusion criteria   - Patients having chest wall skin loss - subcutaneous emphysema - morbid obesity (BMI more than 40) preventing adequate ultrasound evaluation - preexisting pulmonary conditions such as pulmonary surgery or chronic lung disease - haemodynamic instability   Characteristics (participants)  Age [y], mean (±SD)  36.7 (± 13.17)  Male, n (%)  64 (80)  Head injuries, n (%)  60 (75)  Spinal injuries, n (%)  6 (7.5)  Abdominopelvic injuries, n (%)  35 (43.75)  Limb injuries, n (%)  16 (20)  CT chest findings, n (%)  No Pneumothorax: 18 (22.5)  Pneumothorax: 62 (77.5)  Side, Bilateral: 4 (5)  Side, Unilateral: 58 (72.5)  Location, apical: 40 (50)  Location, basal: 12 (15)  Location, medial: 2 (2.5)  Location, lateral: 8 (10) | Participants  N=80 polytraumatized patients  Tests evaluated  Index test: X-Ray & Ultrasonography. Patients were subjected to chest X-ray revealing no pneumothorax. All of those patients underwent thoracic U/S examination using Digital Ultrasonic Imaging System Model Phillips Affiniti 50G and portable Mindray dp20 afterwards. The characteristic ultrasonographic features for detection of pneumothorax were: absence of lung sliding, absence of B lines and identification of the lung point on 2D and M-mode ultrasonography. Occult pneumothorax (OPTX) distribution was described by patient side (unilateral or bilateral), into apical, basal, medial and lateral.  Reference standard: WBCT. After X-ray and thoracic U/S all patients received whole body CT scanning performed by Toshiba Alicson 16 slice within 2 h of admission | Primary outcomes  Diagnostic accuracy for detection of OPTX (chest ultrasonography)  True positive, N=56  False positive, N=2  True negative, N=16  False negative, N=6  Sensitivity, %*  90.32  Specificity, %*  88.89  PPV, %*  96.55  NPV, %*  72.73  Accuracy, %*  90   - *no 95% CI provided   Subgroup analysis ultrasound features  Diagnostic accuracy in detection of B-Lines  True positive, N=56  False positive, N=2  True negative, N=16  False negative, N=6  Sensitivity, %  90.32  Specificity, %  88.89  PPV, %  96.55  NPV, %  72.73  Accuracy, %  90  Diagnostic accuracy in detection of lung sliding  True positive, N=60  False positive, N=4  True negative, N=14  False negative, N=2  Sensitivity, %  96.77  Specificity, %  77.77  PPV, %  93.75  NPV, %  87.5  Accuracy, %  92.5  Diagnostic accuracy in detection of lung point  True positive, N=44  False positive, N=0  True negative, N=18  False negative, N=18  Sensitivity, %  70.97  Specificity, %  100  PPV, %  100  NPV, %  50  Accuracy, %  77.5  Examiners detected signs like lung point and absence of lung sliding, but decision for diagnosis of pneumothorax was mainly made by detecting the absence of B-Lines | Level of evidence  2b  Risk of bias (QUADAS)  Patient selection: ?  Index test: +  Reference standard: ?  Flow and timing: +  Authors’ conclusion  „Bedside thoracic ultrasonography is a simple, rapid and reliable tool with high sensitivity, specificity and accuracy that can be dependable for diagnosis of occult pneumothorax in chest trauma patients.”  Reviewers’ conclusion  Results of the study should be interpreted with caution as the study population is mainly male and young, patients with preexisting pulmonary conditions, which also have a risk for occult PTX, were excluded.  There is an unclear risk of selection bias and reference standard bias as no further information was provided about blinding. The authors report statistical testing but without presenting results. |
| Kozaci (2019)  „Comparison of ultrasonography and computed tomography in the determination of traumatic thoracic injuries.” *American Journal of Emergency Medicine*. 2019;37(5):864-8.  Study design  Diagnostic cross-sectional study  Aim of the study  “In this study, the accuracy of bedside thoracic ultrasonography (TUSG) performed by emergency physicians with patients in the supine position was compared with that of thoracic computed tomography (TCT) for the determination of thoracic injuries due to trauma.”  Setting  Turkey, 2015 – 2018 | Inclusion criteria   - suffered multiple traumas - thoracic trauma was identified on physical examination, and by thoracic computed tomography imaging   Exclusion criteria   - Patients who underwent thoracic computed tomography imaging at other medical centers before referral to the emergency department - pregnant patients   Characteristics  Age [y], mean ± SD  38 ± 20  Male, n (%)  64 (79)  GCS, n (%)  14–15: 73 (89)  9–13: 2 (3)  3–8: 7 (9) | Participants  N=81 patients, 76 patients analysed  Tests evaluated  Index test: Thoracic ultrasonography, performed following a physical examination by an emergency physician to identify thoracic injuries. Pneumothorax was diagnosed if: B lines, lung sliding and lung pulse were absent (examination with linear probe). The presence of hemothorax was considered when the anechoic area was detected in the pleural area with a convex probe. Pulmonary contusion was diagnosed if B and C lines were detected with the linear probe and hepatization and parenchymal disruption were detected with the convex probe.  Reference standard: Thoracic computed tomography, performed after the ultrasonography examination was completed. | Primary outcomes  Diagnostic accuracy for detection of pneumothorax (thoracic ultrasonography)  N=76  Sensitivity [%]  86  Specificity [%]  97  AUC (95% CI)  0.912 (0.820–1.000)  Diagnostic accuracy for detection of hemothorax (thoracic ultrasonography)  Sensitivity [%]  45  Specificity [%]  98  AUC (95% CI)  0.717 (0.567–0.867)  Diagnostic accuracy for detection of pulmonary contusion (thoracic ultrasonography)  Sensitivity [%]  63  Specificity [%]  91  AUC (95% CI)  0.769 (0.648–0.889) | Level of evidence  2b  Risk of bias (QUADAS)  Patient selection: +  Index test: ?  Reference standard: ?  Flow and timing: -  Authors’ conclusion  “In conclusion, ultrasound was found to be highly specific but only moderately sensitive for the identification of thoracic injuries.”  Reviewers’ conclusion  Unclear risk of bias due to missing information about blinded examiners to the results of index and reference tests. Additionally 5 participants were not included in analysis; reasons for this are not reported. This leads to risk of bias in flow and timing. |
| Leblanc (2014)  “Early lung ultrasonography predicts the occurrence of acute respiratory distress syndrome in blunt trauma patients”. *Intensive Care Medicine.* 2014;40(10):1468-74.  Study design  Diagnostic cross-sectional study  Aim of the study  “We hypothesized that early assessment of lung contusion extent using lung ultrasonography (LUS) can predict the occurrence of acute respiratory distress syndrome (ARDS) in blunt trauma patients.”  Setting  France, 2010-2011 | Inclusion criteria   - Patients with multiple blunt trauma were enrolled in the study if one of the physicians trained in lung ultrasonography (i.e., with an experience of more than 30 LUS in trauma patients) was present.   Exclusion criteria  n.r.  Characteristics (participants)  Age [y], mean ± SD  35 ± 16  Male, n (%)  32 (71)  ISS, median (IQR)  34 (25–48)  GCS , mean (± SD)  11 ± 4 | Participants  N=45 multiple trauma patients  Tests evaluated  Index test 1: combined physical examination and chest radiography  Index test 2: Lung ultrasonography (LUS) was performed after completion of clinical examination by an anesthesiologist trained for LUS blinded to the clinical examination and chest radiography (CXR) results.  Reference standard: CT | Primary outcomes  Diagnostic accuracy for detection of pneumothorax (lung ultrasonography)  AUC-ROC (95% CI)*  0.81 (0.50–1.00) vs. 0.74 (0.48–1.00), p=0.24^§^  Diagnostic accuracy for detection of lung contusion  AUC- ROC (95% CI)*  0.88 (0.76–1.00) vs. 0.69 (0.47–0.92), p<0.05  Diagnostic accuracy for detection of hemothorax  AUC- ROC (95% CI)*  0.84 (0.59–1.00) vs. 0.73(0.51–0.94), p<0.05  *compared to reference standard  ^§^no significant difference between the two diagnostic modalities for pneumothorax diagnosis  Index test 1  *detection of pneumothorax*  Sensitivity [%] (95% CI)  50 (NR)  Specificity [%] (95% CI)  92 (NR)  *detection of hemothorax*  Sensitivity [%] (95% CI)  52 (NR)  Specificity [%] (95% CI)  80 (NR)  *detection of pulmonary contusion*  Sensitivity [%] (95% CI)  78 (NR)  Specificity [%] (95% CI)  57 (NR)  Index test 2  *detection of pneumothorax*  Sensitivity [%] (95% CI)  53 (NR)  Specificity [%] (95% CI)  95 (NR)  *detection of hemothorax*  Sensitivity [%] (95% CI)  60 (NR)  Specificity [%] (95% CI)  99 (NR)  *detection of pulmonary contusion*  Sensitivity [%] (95% CI)  90 (NR)  Specificity [%] (95% CI)  87 (NR) | Level of evidence  2b  Risk of bias (QUADAS)  Patient selection: +  Index test: +  Reference standard: +  Flow and timing: +  Authors’ conclusion  “(…)lung ultrasonography on admission identifies patients at risk of developing ARDS after blunt trauma. In addition, lung ultrasonography allows rapid and accurate diagnosis of common traumatic thoracic injuries.”  Reviewers’ conclusion  Risk of bias is low, but the sample size is small. |
| Ojaghi Haghighi (2014)  “Ultrasonographic diagnosis of suspected hemopneumothorax in trauma patients.” *Trauma Monthly.* 2014;19(4):e17498.  Study design  Diagnostic cross-sectional study  Aim of the study  “The aim of this study was to evaluate the sensitivity and specificity of ultrasonography in the diagnosis of pneumothorax and hemothorax in comparison with portable CXR and CT-Scan.”  Setting  Iran, 2013 | Inclusion criteria   - Patients with severe multiple trauma who were suspected of having chest injuries, and who had indications for a chest CT-scan according to ATLS algorithms   Exclusion criteria   - Patients who underwent a tube thoracostomy, before they had an opportunity to have an ultrasound due to their unstable clinical situation, or for any other reason, such as a lack of access to ultrasound at the time of admission, were excluded from the study.   Characteristics  Age [y], mean ± SD  n.r  Male, n (%)  124 (82.66)  ISS  n.r | Participants  N=150 patients  Tests evaluated  Index test 1: ultrasonography  Index test 2: portable chest radiography  Reference test: CT  Notes   - Examination findings included: chest pain, tenderness over the ribs, decreased lung sounds or chest percussion, subcutaneous emphysema, or any sign of trauma such as abrasions and/or bruises.   Patients were evaluated according to the ATLS algorithm, and examination findings were recorded following initial evaluations, an emergency medicine specialist performed chest ultrasonography to detect pneumothorax and hemothorax. | Primary outcomes  Diagnostic accuracy for detection of pneumothorax (ultrasonography)  True positive: 50  True negative: 98  False positive: 0  False negative: 2  Sensitivity [%]  96.15  Specificity [%]  100  PPV [%]  100  NPV [%]  98  Diagnostic accuracy for detection of hemothorax (ultrasonography)  True positive: 39  True negative: 101  False positive: 2  False negative: 8  Sensitivity [%]  82.97  Specificity [%]  98.05  PPV [%]  95.12  NPV [%]  92.66  Diagnostic accuracy for detection of pneumothorax (portable CXR)  True positive: 18  True negative: 96  False positive: 2  False negative: 34  Sensitivity [%]  34.61  Specificity [%]  97.95  PPV [%]  90  NPV [%]  73.84  Diagnostic accuracy for detection of hemothorax (portable CXR)  True positive: 12  True negative: 98  False positive: 5  False negative: 35  Sensitivity [%]  25.53  Specificity [%]  95.14  PPV [%]  70.58  NPV [%]  73.68 | Level of evidence  2b  Risk of bias (QUADAS)  Patient selection: ?  Index test: +  Reference standard: +  Flow and timing: +  Authors’ conclusion  “Ultrasonography sensitivity and specificity for diagnosis of hemopneumothorax was high. The sensitivity of portable CXR was low despite its high specificity for the detection of hemothorax and pneumothorax”  Reviewers’ conclusion  There is an unclear risk of bias due to missing information regarding patient selection. |
| Zanobetti (2018) *(already extracted for 2.121)*  „Chest Abdominal‑Focused Assessment Sonography for Trauma during the primary survey in the Emergency Department: the CA‑FAST protocol” *European Journal of Trauma Emergency Surgery* (2018); 44: 805-810  Study design  Diagnostic cross-sectional study  Aim of the study  „(…)we developed a new protocol (CA-FAST, Chest Abdominal FAST) that integrates the detection of LCs in the E-FAST examination which can be performed during the primary survey. The aim of this study was to evaluate the feasibility and the diagnostic performance of CA-FAST examination when compared to the gold standard, thoracoabdominal CT.”  Setting  Italy, 2012-2013 | Inclusion criteria   - Adult trauma patients presenting to the ED - Underwent a thoracoabdominal CT scan were enrolled if a CA-FAST examination was previously performed   Exclusion criteria  n.r.  Characteristics (participants)  Age [y], mean (±SD)  46 (±20)  Male, n (%)  467 (75)  ISS, mean (±SD)  16 (±12) | Participants  N=601 trauma patients  Tests evaluated  Index test: Chest Abdominal Focussed Assessment Sonography for Trauma (CA-FAST) protocol, CA-FAST protocol consisted of a combined ultrasonographic evaluation of chest and abdomen in order to detect the presence of the following ultrasonographic patterns: pneumothorax (PTX), pleural effusion (PE), lung contusion (LC), pericardial and intraperitoneal effusion. The full examination consisted of 8 chest scans and 4 abdominal scans acquired with the patient in obligated supine position due to spinal boards and cervical collars. Chest US was performed by a 4- to 8-MHz linear probe or a 3.5- to 5-MHz curved array probe. Performance of CA-FAST by 12 emergency physicians. The abdominal US was performed by 5-MHz curved array probe using the standard 4-views.  Reference standard: thoracoabdominal CT scan, scans were enrolled if a CA-FAST examination was previously performed; CT scan was either required or not at discretion of the emergency physician (EP), independently of patient’s participation to the study. | Primary outcomes  Diagnostic accuracy for detection of pneumothorax (chest ultrasound)  Sensitivity, % (95% CI)  84 (77–89)  Specificity, % (95% CI)  98 (96–99)  PPV, % (95% CI)  93 (87–96)  NPV, % (95% CI)  95 (93–97)  +LR, % (95% CI)  39 (21–73)  −LR, % (95% CI)  0.2 (0.1–0.2)  Accuracy, %, (95% CI)  95 (91–97)  Diagnostic accuracy for detection of pleural effusion(chest ultrasound)  Sensitivity, % (95% CI)  82 (74–88)  Specificity, % (95% CI)  97 (95–98)  PPV, % (95% CI)  87 (79–92)  NPV, % (95% CI)  95 (93–97)  +LR, % (95% CI)  25 (16–41)  −LR, % (95% CI)  0.2 (0.1–0.3)  Accuracy, %, (95% CI)  94 (87–95)  Diagnostic accuracy for detection of lung contusion(chest ultrasound)  Sensitivity, % (95% CI)  59 (51–66)  Specificity, % (95% CI)  98 (96–99)  PPV, % (95% CI)  92 (86–96)  NPV, % (95% CI)  86 (82–89)  +LR, % (95% CI)  29 (15–55)  −LR, % (95% CI)  0.4 (0.4–0.5)  Accuracy, %, (95% CI)  87% (95% CI 85–92)  Other outcomes  Time [min] for examination , average (±SD)  CA-FAST: 7 (±3)  Chest Ultrasound only: 4 (±2)  Abdominal FAST only: 3 (±1)  *Note: Outcomes for 4-view fast are reported under 2.121* | Level of evidence  3b  Risk of bias (QIADAS)  Patient selection: ?  Index test: +  Reference standard: +  Flow and timing:?  Authors’ conclusion  „In summary CA-FAST protocol, performed in the emergency setting, showed important advantages: It is a noninvasive, rapid, ionizing radiation-free and an easily repeatable method; in trauma patients it allows to accurately and immediately detect diagnostic information and ultrasonographic patterns of severe injury. Moreover, the addition of four chest scans and the research of LCs did not cause a delay in the diagnosis.(…) CA-FAST protocol could represent an integrative tool of traditional CT scan in the management of trauma patients; it should be used as the initial investigation, during the primary survey, sending to further diagnostic studies only patients not clearly classified.”  Reviewers’ conclusion  There is an unclear risk of patient selection bias in this study as no further information about exclusion criteria was provided. Missing information about time intervals between the examinations lead to the conclusion of unclear risk for bias in the flow and timing. |
| +: low risk; –: high risk; ?: unclear risk; CA-Fast: Chest-abdominal Focused Assessment Sonography for Trauma; CI: Confidence Interval; CT: Computer Tomography, E-Fast: extended Focused Abdominal Sonography for Trauma; EM_ Emergency Medicine; GCS: Glasgow Coma Scale; IQR: Interquartile Range; ISS: Injury Severity Score; LC: Lung Contusion; OR: Odds Ratio; SD: Standard Deviation; PPV: Positive Predictive Value; NPV: Negative Predictive Value; AUC: Area under the curve; +LR: Positive Likelihood Ratio; -LR: Negative Likelihood Ratio; adj.: adjusted; d: days; m: months; y: years; min: minutes; POCUS: Point of Care Ultrasound; PTX: Pneumothorax; RUSH: Rapid Ultrasound in Shock and Hypotension; SBP: systolic Blood Pressure; | | | | |

##### X-ray of the thorax as an alterantive to immediate CT thorax.

| Study: Reference, aim, design, setting | Participants: selection criteria, characteristics | N Participants;  Intervention (IG) vs. Control group (CG) | Main outcomes | Assessment: LoE, risk of bias; Conclusions |
| --- | --- | --- | --- | --- |
| Bolteho Finho (2015)  “Complementary exams in blunt torso trauma. Perform only radiographs and fast: is it safe?”. *Rev. Col. Bras. Cir.* 2015; 42(4): 220-223  Study design  Diagnostic cross-sectional study  Aim of the study  „(…) it was decided to investigate the extent to which radiological examinations of primary trauma assessments (pelvic and chest X-rays and FAST - focused abdominal sonography trauma) are sufficient in indicating the best approach for the initial care of polytrauma patients.”  Setting  Brazil, 2013-2014 | Inclusion criteria   - Blunt trauma patients   Exclusion criteria   - n.r.   Characteristics  Age [y], average  33  Male, n (%)  n.r.  Revised Trauma Score, mean  6.98  GCS, mean  12 | Participants  N=74 patients  Tests evaluated  Index test: set of three examinations (chest X-ray, pelvis X-ray and FAST). The set of examinations was performed on the included blunt trauma patients to identify traumatic injuries.  Reference standard 1: CT scan of the torso of the same patients was used as reference standard  Reference standard 2: if patients did not have CT scans clinical observation during hospitalization was replaced as reference standard (7 days for those who were intubated, 48h for patients who were conscious but required hospitalization for some other reason (e.g. orthopedic fracture), 12 hours for those who remained in hospital for only the minimum observation time) | Primary outcomes  Diagnostic accuracy for screening for significant injuries  True positive, N=27  False positive, N=3  True negative, N=41  False negative, N=3  Sensitivity, %*  90  Specificity, %*  93  NPV, %*  93  PPV, %*  89  *No 95% CI provided | Level of evidence  3b  Risk of bias (QUADAS)  Patient selection: -  Index test: -  Reference standard: -  Flow and timing: -  Authors’ conclusion  „We conclude [sic] that CT can be used selectively in cases of altered clinical examinations or when the patient shows alterations in these requested examinations. A full-body CT scan therefore does not [sic] need be used for an initial diagnosis for all polytrauma patients, which is in line [sic] with Brazilian reality, the reality of a developing country which is seeking to reduce medical costs wherever possible.”  Reviewers’ conclusion  Results of the study should be interpreted with caution as the methodology lacks essential information about recruitment, sampling, randomization, blinding and drop-out patients. There are risks of bias concerning patient selection and index test. Besides that, not all patients received the same reference test which causes risk of bias in reference standard and flow and timing.  In general, it is debatable that intubated trauma patients did not receive an adequate initial diagnostic imaging with CT scans, but were observed only. |
| +: low risk; –: high risk; ?: unclear risk; CI: Confidence Interval; IQR: Interquartile Range; OR: Odds Ratio; RR: Relative Risk; SD: Standard Deviation; PPV: Positive Predictive Value; NPV: Negative Predictive Value; AUC: Area under the curve; +LR: Positive Likelihood Ratio; -LR: Negative Likelihood Ratio; adj.: adjusted; d: days; m: months; y: years; min: minutes | | | | |

##### Timing and indications for whole body CT, trauma specific protocol.

| Study: Reference, aim, design, setting | Participants: selection criteria, characteristics | N Participants;  Intervention (IG) vs. Control group (CG) | Main outcomes | Assessment: LoE, risk of bias; Conclusions |
| --- | --- | --- | --- | --- |
| Bieler (2020)  „Why do some trauma patients die while others survive? A matched-pair analysis based on data from Trauma Register DGU R”. *Chinese Journal of Traumatology* 2020; 23(4): 224-232  Study design  Case-control study  (Trauma Register DGU®)  Aim of the study  „The aim of this study was to use a matched-pair analysis in order to identify factors that influence the mortality of severely injured patients (ISS≥16). The limitations of retrospective register studies must be kept in mind.”  Setting  Germany, 2009-2014 | Inclusion criteria   - patients who received primary care were included - ISS≥16   Exclusion criteria   - Patients who were transferred from another hospital or were transferred to another hospital at an early stage - younger than 16 years of age and older than 55 in order to minimize age-related factors that could influence mortality - Patients with American Society of Anaesthesiologists Classification (ASA) scores 5 and 6   Characteristics  Age [y], mean (SD)  SV: 36.8 ± 12.4  NSV: 36.9 ± 12.4  Male, n (%)  SV: 535 (81.4)  NSV: 535 (81.4)  ISS, mean (SD)  SV: 30.7 ± 8,7  NSV: 30.9 ± 9,4  GCS at scene of accident, mean (SD)  SV: 7.4 ± 4.3  NSV: 6.6 ± 4.5 <0.001 | Participants  N=1,314 patients  Study groups  SV: Survivor group, 657 patients  NSV: Non-Survivor, 657 patients  Matching criteria   - Four age groups were created (16-25, 26-35, 36-45 and 46-55 years). - Two categories, i.e. American Society of Anaesthesiologists Classification System (ASA) 1, 2 and ASA 3, 4 were created to match pre-traumatic states of health, and the partners were allocated to these categories. - Patients were matched on the basis of the AIS for four relevant body regions in order to take into account the influence of injury patterns. - After the data transformation of the respective matched pair criteria into a numerical code, the matching of non-survivor to a survivor was done by the authors RL and DB in four-eye principle.   Two homogeneous groups were formed. | Primary outcomes  Factors associated with survival or nonsurvival (Procedures received)  WBCT, n (%)  SV: 597 (91.1)  NSV: 565 (86.4)  p=0.006  cCT, n (%)  SV:650 (98.9)  NSV:622 (94.7)  p<0.001  FAST, n (%)  SV: 525 (80.2)  NSV: 520 (79.5)  p=0.77  Conventional X-ray diagnostic, n (%)  SV: 258 (39.4)  NSV: 232 (35.5)  p=0.14 | Level of evidence  3b  Risk of bias  Selection of participants: +  Assessment: +  Confounding factors: +  Statistical analysis: -  Authors’ conclusion  „The results of this study show that there are significant factors that predict or influence the mortality of severely injured patients. On the basis of paraclinical values as hemoglobin level and base excess, bleeding patients in particular are likely to have an unfavorable outcome. The mechanism of injury also appears to have an influence on the likelihood of survival. In this study a car accident is associated with a significant better outcome concerning mortality and a fall of >3 m is survived significantly less. Factors that other studies have found to exert an influence, such as care level and the length of the emergency room stay, did not make a significant difference in our study.”  Reviewers’ conclusion  Results need to be interpreted with caution due to the retrospective design of the study.. The imitations due to the case-control design were minimized by the matching procedure. |
| Lang (2017)  „The role of whole-body computed tomography in the diagnosis of thoracic injuries in severely injured patients - a retrospective multi-centre study based on the trauma registry of the German trauma society (TraumaRegister DGU^R^).” *Scandinavian Journal of Trauma, Resuscitation & Emergency Medicine.* 2017;25(1):82.  Study design  Comparative registry study  (TraumaRegister DGU®)  Aim of the study  We conducted a retrospective analysis of the trauma registry of the German Trauma Society (TraumaRegister DGU®) in order to assess the number of diagnosed thoracic injuries before and after the introduction of WBCT as a standard imaging modality and to investigate whether the trauma scan led to a change in patient outcomes.  Setting  Germany, 2002-2012 | Inclusion criteria   - all cases of patients who were admitted to the trauma room with an ISS ≥9 - Only patients who underwent primary treatment at a regional (Level II) or supraregional trauma centre (Level I) (as defined by the TraumaRegister DGU®) were included - continuous documentation over a period of at least five consecutive years was required   Exclusion criteria   - Patients who did not undergo immediate surgery or were not admitted to ICU - The patients who underwent an imaging procedure in the year in which the trauma scan was introduced for routine use (N=2981; 18.0%) were not included in this study because of a wide variety of implementation rates   Characteristics  Age [y], mean (95% CI)  IG: 45.7 (45.2–46.1)  CG: 43.0 (42.5–43.6)  Male, n (%)  IG: 6248 (73.0)  CG: 3677 (73.5)  Patients with blunt trauma [%], mean (95% CI)  IG: 94.5 (94.2–95.0)  CG: 94.8 (94.2–95.2)  GCS, mean (95% CI)  IG: 11.1 (11.0–11.2)  CG: 11.0 (10.8–11.1)  ISS, mean (95% CI)  IG: 24.5 (24.2–24.7)  CG: 23.9 (23.5–24.2)  Type of thoracic injuries  Injury to the lung parenchyma, % (n.r.)  IG: 5.9 (5.4–6.4) CG: 12.6 (11.7–13.5)  Pulmonary contusion, % (n.r.)  IG: 28,7 (27.7–29.7) CG: 18,5 (17.4–19.6)  Pneumothorax, % (n.r.)  IG: 21.6 (20.7–22.5) CG: 17.3 (16.3–18.4)  Tension pneumothorax, % (n.r.)  IG: 2.5 (2.2–2.8) CG: 2.9 (2.4–3.4)  Haemothorax, % (n.r.)  IG: 14.0 (13.3–14.7) CG: 15.6 (14.6–16.6)  Multiple rib fractures and flail chest, % (n.r.)  IG: 21.6 (20.7–22.5) CG: 10.6 (9.7–11.4)  Arterial injury (thorax), % (n.r.)  IG: 1.5 (1.2–1.8) CG: 1.5 (1.2–1.8)  Diaphragmatic injury, % (n.r.)  IG: 1.1 (0.9–1.3) CG: 1.0 (0.7–1.3)  Thoracic spine injury ≥AIS 2, % (n.r.)  IG: 13.2 (12.5–13.9) CG: 10.9 (10.0–11.8)  Thoracic spinal cord injury, % (n.r.)  IG: 1.9 (1.6–2.2) CG: 1.7 (1.3–2.1)  Cardiac injury, % (n.r.)  IG: 0.5 (0.4–0.7) CG: 0.4 (0.2–0.6) | Participants  N=16,545 trauma patients  Study groups  IG: trauma scan/ WBCT group, n=8,559  CG: traditional diagnostic imaging/ pre-WBCT group, n=5,002  Study interventions   - For cases in which the box for Whole-body CT in the TraumaRegister DGU® data collection form was checked, we assumed that a whole-body trauma scan was performed as a primary diagnostic procedure. The TR-DGU defines WBCT as a combination of CT studies that produce images (or slices) of the body in a continuous manner and cover at least the region from the skull base to the pelvis.   The other diagnostic approach consists of traditional imaging that involves conventional radiography of the cervical spine, the chest and the pelvis, often followed by focused CT (e.g. cranial CT). | Primary outcomes  Length of stay in the trauma room [min], mean (95% CI)  IG: 64 (63.0 – 65.0)  CG: 78 (76.0–79.0)  Length of ICU stay [d], mean (95% CI)  IG: 9.7 (9.4–10.0) CG: 10.8 (10.5–11.2), p=nr  Length of intubation/ventilation [d], mean (95% CI)  IG: 5.6 (5.4–5.8) CG: 6.9 (6.6–7.2)  Length of hospital stay [d], mean (95% CI)  IG: 23.3 (22.7–23.8) CG: 26.2 (25.8–26.9)  Ventilator-free days [d], mean (95% CI)  IG: 20.8 (20.6–1.1) CG: 19.8 (19.4–20.1)  24-h mortality [%], mean (95% CI)  IG: 8.2 (7.6–8.8) CG: 8.9 (8.1–9.7)  Hospital mortality [%], mean (95% CI)  IG: 15.6 (14.9–16.4) CG: 15.5 (14.5–16.5)  Organ failure [%], mean (95% CI)  IG: 43.9 (42.7–45.1) CG: 43.5 (42.0–45.0)  Pulmonary failure [%], mean (95% CI)  IG: 22.2 (21.2–23.2) CG: 26.2 (24.9–27.6)  Multi-organ failure [%] mean (CI)  IG: 26.9 (25.8–27.9) CG: 26.5 (25.2–27.8) | Level of evidence  2b  Risk of bias  Selection bias: –  Performance bias: ?  Attrition bias: +  Detection bias: +  Authors’ conclusion  „Following the replacement of traditional imaging (conventional radiography and focused CT) by WBCT as the standard imaging modality in the trauma room setting, a higher number of thoracic injuries were detected. The majority of these cases, however, were minor injuries requiring no immediate treatment. There was no change in the clinical management of the thoracic injuries investigated here. During the period from 2002 to 2012, the routine use of the trauma scan did not improve survival in the non-selected patient population (ISS = 9). WBCT, however, led to a relevant reduction in the time spent in the trauma room (i.e. from 78 to 64 min).“  Reviewers’ conclusion  Even though risk of attrition and detection bias is low, there might be a moderate overall risk of bias due to high risk of bias regarding selection of participants and unclear performance bias. |
| Palm (2018)  “Changes in trauma management following the implementation of the whole-body computed tomography: a retrospective multicentre study based on the trauma registry of the German Trauma Society (TraumaRegister DGU®)”.  Study design  Comparative registry study  (TraumaRegister DGU®)  Aim of the study  „The objective of our retrospective multi-centre study was to analyse data from the TraumaRegister DGU® to assess whether the introduction of the trauma scan led to changes in terms of the number of injuries detected, the body parts affected, the indication-dependent management of patients following the completion of trauma room procedures, and patient outcomes and thus to determine whether trauma room processes before WBCT were different from those after the introduction of the trauma scan as a standard imaging modality.” *European Journal of Trauma Emergency Surgery* 2018; 44:759–766  Setting  Germany, 2002-2013 | Inclusion criteria   - Patients who were admitted to the trauma room with an Injury Severity Score (ISS) ≥9 and who either underwent surgery or were transferred to the intensive care unit (ICU) following management in the trauma room or died in the trauma room. - Patients who underwent primary treatment at a regional (level II) or supraregional (level I) trauma centre were included.   Exclusion criteria  The year in which the trauma scan was introduced was excluded from analysis since this year was usually a period of transition associated with a mixture of both imaging approaches.Characteristics  Age [y], median (95% CI)  IG: 46.6 (45.9–47.2)  CG: 43.0 (42.1–43.9)  Male, % (95% CI)  IG: 72.2 (71.6–72.8)  CG: 72.7 (72.1–73.3)  ISS, mean (95% CI)  IG: 23.9 (23.4–24.3)  CG: 23.7 (23.1–24.3) | Participants  N=16,928 trauma patients  Study groups  IG: whole-body computed tomography scan (WBCT) group, patients underwent whole-body multi-slice CT as the primary diagnostic imaging modality; N=11,307  CG: before WBCT was introduced (Pre-WBCT) group, patients in this group underwent diagnostic procedures such as conventional radiography, abdominal ultrasound and focused CT; N=5,621  Study intervention   - Up to 3 years before (pre-WBCT group) and up to 3 years after the introduction of the trauma scan (WBCT group) as a standard imaging procedure were analysed and compared - A maximum variation of 30% in both the pre-WBCT and the WBCT group provided the basis for decision. In addition, there had to be an increase in the WBCT rate by at least 50% or to at least 60% in the year following the introduction of WBCT when compared to the year preceding the introduction of the trauma scan. | Primary outcomes  Mortality, % (95% CI)  IG: 15.9 (15.4–16.4)  CG: 15.7 (15.2–16.3)  Mortality according to RISC II, %  IG: 15.2  CG: 15.7  Number of diagnoses, mean (95% CI)  IG: 5.1 (5.0–5.2)  CG: 4.6 (4.5–4.7)  Patients underwent surgery immediately after completion of treatment in the trauma room, % (95% CI)  IG: 39.1 (38.3–39.9)  CG: 44.5 (43.7–45.3)  Patients who required emergency surgery or died during trauma room management, % (95% CI)  IG: 5.1 (4.7–5.5)  CG: 6.8 (6.4–7.2)  Patients directly transferred to the ICU, % (95% CI)  IG: 54.4 (53.6–55.2)  CG: 46.2 (45.4–47.0)  Total time spent in the trauma room [min], mean (95% CI)  IG: 63.6 (62.0–65.1)  CG: 77.9 (75.7–80.2)  Length of ICU stay [d], mean (95% CI)  IG: 8.9 (8.6–9.3)  CG: 10.6 (10.0–11.2)  Length of hospital stay [d], mean (95% CI)  IG: 21.6 (20.9–22.3)  CG: 25.3 (24.1–26.4) | Level of evidence  2b  Risk of bias  Selection bias: +  Performance bias: ?  Attrition bias: +  Detection bias: +  Authors’ conclusion  „Our study shows that the trauma scan is not superior to a combination of conventional radiography, ultrasound and focused CT in terms of mortality in a non-selected population of patients (ISS ≥9). Against this background, more importance should be placed on decision trees that are independent of imaging modalities and allow trauma team leaders to decide on an individual basis whether a patient is a candidate for a trauma scan. Our study also showed, however, that the introduction of the trauma scan as a standard imaging modality led to a relevant reduction in almost all trauma room processes and enabled trauma teams to make faster treatment decisions. We observed an increase in the number of diagnoses per patient. Since we were also able to prove that the introduction of the trauma scan was associated with a relevant decrease in the length of ICU stay and hospital stay, trauma teams should adhere to current practices and procedures until the aforementioned decision trees are available.”  Reviewers’ conclusion  There is an unclear risk of performance bias in this study as it is unclear if the knowledge about the diagnostic procedure may have influenced the care. The study population overlaps significant with the one analyzed in Lang (2017), which should be considered during interpretation of both studies results. The effect size might be overestimated due to double analysis of the same population. |
| Sierink (2016)  “Sierink, J.C., et al., Immediate total-body CT scanning versus conventional imaging and selective CT scanning in patients with severe trauma (REACT-2): a randomised controlled trial.” *Lancet*, 2016. 388(10045): p. 673-83.  Study design  Randomised controlled trial  Aim of the study  „We undertook a randomised clinical trial (REACT-2) to examine the eff ect of immediate total-body CT scanning as part of the primary assessment of patients with severe trauma on in-hospital mortality, and compared it with that of the standard work-up of conventional imaging supplemented with selective CT scanning.”  Setting  The Netherlands and Switzerland, 04.2011 -01.2014 | Inclusion criteria   - Trauma patients with the presence of life-threatening vital problems, at least one of the following: respiratory rate ≥30 min of ≤10/min; pulse ≥120/min; systolic blood pressure ≤100 mmHg; estimated exterior blood loss ≥500 ml; Glasgow Coma Score ≤13; Abnormal pupillary reaction onsite. - patients with one of the following clinically suspicious diagnoses: flail chest, open chest or multiple rib fractures; severe abdominal injury; pelvic fracture; unstable vertebral fractures/spinal cord compression; fractures from at least two long bones - patients with one of the following injury mechanisms: fall from height (>3 m/>10 ft); ejection from the vehicle; death occupant in same vehicle; severely injured patient in same vehicle; wedged or trapped chest/abdomen.   Exclusion criteria   - known age <18 years - known pregnancy - referred from another hospital - clearly low-energy trauma with blunt injury mechanism - penetrating injury in 1 body region (except gunshot wounds) as the clearly isolated injury - any patient who is judged to be too unstable to undergo a CT scan and requires (cardiopulmonary) resuscitation or immediate operation because death is imminent according to the trauma team leader in mutual agreement with the other leading care givers.   Characteristics  Age [y], median (IQR)  IG: 42 (27–59) vs. CG: 45 (26–59)  Male sex, n (%)  IG: 413 (76) vs. CG: 411 (76)  In hospital GCS, median (IQR):  IG: 13 (3–15) vs. CG: 13 (3–15)  Abbreviated Injury Scale ≥3, n (%)  Head: IG: 247 (46) vs. CG: 218 (40)  Chest: IG: 229 (42) vs. CG: 206 (38)  Abdomen: IG: 49 (9) vs. CG: 67 (12)  Arms, legs, hand, and feet: IG: 150 (28) vs. CG: 154 (28)  ISS, median (IQR):  IG: 20 (10–29) vs. CG: 542 19 (9–29)  Patients with polytrauma, n (%)  IG: 362 (67%) vs. CG: 331 (61)  Patients with traumatic brain injury, mean (SD):  IG: 178 (32.9) vs. CG: 151 (27.9) | Participants  N=1,403 patients randomized, 1083 analyzed  Study groups  IG: Total-body CT; following a two-step acquisition (from vertex to pubic symphysis) without gantry angulations:starting with a non-enhanced CT of the head and neck with arms alongside the trunk. The second scan covered the chest, abdomen, and pelvis. The second scan was split-bolus intravenous contrast imaging immediately after raising the arms alongside the head. CT scanners were all 64-slice multidetector row CT scanners; N=541  CG: standard work-up; according to ATLS guidelines with chest and pelvic radiographs and focused assessment with sonography in trauma. During the secondary survey, a selective CT scan could be made from individual body regions, with segmented acquisition of the respective body regions; N=542  Note  ITT analysis  ‡Patients who died in the emergency department (six [1%] of 541 patients in the total-body CT group vs four [1%] of 542 in the standard work-up group) and those with incomplete follow-up for radiation exposure (15 [3%] vs seven [1%]) were excluded.  §Packed cells, thrombocytes, or plasma.  ¶Excluded patients who died during the initial admission (86 patients in the total-body CT group and 85 in the standard work-up group). \|\|Excluded patients with incomplete follow-up for readmissions (60 in the total-body CT group and 45 in the standard work-up group).  **One other serious adverse event occurred in a patient who was excluded after random allocation. | Primary outcomes  In-hospital mortality, n (%)  IG: 86 (16%) vs. 85 (16%), p=0.92  24-h mortality, n (%)  IG: 43 (8) vs. CG 33 (6), p=0.23  30-day mortality, n (%)  IG: 81 (17) vs. CG: 78 (16), p=0.69  Complications, n (%)  IG: 129 (24) vs. CG: 124 (23), p=0.73  Serious adverse events (safety endpoint)**, n (%)  IG: 3 (1) vs. 1 (<1), p=0.37  Blood transfusions in hospital, n (%) §  IG: 147 (27) vs. 150 (28), p=0.91  Length of stay at ICU [d] , median (IQR)¶  IG: 3 (1–8) vs. CG: 3 (1–8),p=0.83  Ventilator use [d], median (IQR)  IG: 2 (1–5) vs. CG: 1 (1–6), p=0.78  Time to end of imaging [min], median (IQR)  IG: 30 (24–40) vs. CG: 37 (28–52); p<0.0001  Time spend in the trauma room [min], median (IQR)  IG: 63 (47–102) vs. CG: 72 (50–109); p=0.067  Readmission within 6 months, n (%)\|\|  IG: 67 (17) vs. CG. 44 (11), p=0.01  Subgroup analysis patients with polytrauma  In-hospital mortality, n (%)  IG: 81 (22) vs. CG: 82 (25), p=0.46  24-h mortality, n (%)  IG: 41 (11) vs. CG 33 (10), p=0.56  30-day mortality, n (%)  IG: 76 (23) vs. 75 (24), p=0.69  Subgroup analysis patients with severe TBI  In-hospital mortality, n (%)  IG: 68 (38) vs. CG 66 (44), p=0.31  24-h mortality, n (%)  IG: 37 (21) vs. CG: 27 (18), p=0.51  30-day mortality, n (%)  IG: 66 (39) vs. CG: 60 (41), p=0.65 | Level of evidence  1b  Risk of bias  Selection bias: +  Performance bias: –  Attrition bias: +  Detection bias: +  Authors’ conclusion  “Diagnosing patients with an immediate total-body CT scan does not reduce in-hospital mortality compared with the standard radiological work-up. Because of the increased radiation dose, future research should focus on the selection of patients who will benefit from immediate total-body CT.”  Reviewers’ conclusion  The study was a well conducted RCT achieving the sample size necessary to detect differences in mortality. Physicians and patients could not be blinded to the intervention. |
| Topp (2015)  “Radiologic diagnostic procedures in severely injured patients - is only whole-body multislice computed tomography the answer?”. *International Journal of Emergency Medicine* 2015; 8:3  Study design  Comparative registry study  (TraumaRegister DGU®)  Aim of the study  „The objective of this study was to compare a protocol that uses whole-body multi-sclice CT (WB-MSCT) as the first and only diagnostic tool to a protocol that uses conventional radiographs prior to WB-MSCT regarding a) duration of the initial treatment phase in the resuscitation room, b) length of stay in the intensive care unit (ICU), c) ventilation days, d) length of hospital stay, and e) mortality.”  Setting  Germany, 1993-2009 | Inclusion criteria   - primary admitted patients with an ISS of ≥16 - documented times of conventional radiographs and WB-MSCT diagnosis   Exclusion criteria   - Patients with penetrating injuries   Characteristics  Age [y], mean (SD)  IG: 45.1 (±19.8)  CG: 45.5 (±20)  p=0.429  Male, n (%)  IG: 2,932 (72.8)  CG: 2,937 (73.5)  p=0.497  ISS, mean (%)  IG: 29.9 (±12.6)  CG: 29.9 (±12.3)  p=0.913  AIS head, n (%)  IG: 2,338 (57.7)  CG: 2,220 (55.1), p<0.05  AIS thorax, n (%)  IG: 2,603 (64.2)  CG: 2,558 (63.5), p=0.501  AIS abdomen, n (%)  IG: 877 (21.6)  CG: 944 (23.4), p<0.05  AIS extremities, n (%)  IG: 1,543 (38.1)  CG: 1,667 (41.4) p<0.01 | Participants  N=8,020 patients  Study groups  IG: group received initial WB-MSCT, mostly with the scanner located in the resuscitation room; N=4,025  CG: group received conventional radiographs prior to WB-MSCT, N=3,995  Study interventions  Both groups also received an initial FAST ultrasound. | Primary outcomes  Mortality, n (%)  IG: 746 (18.4)  CG: 732 (18.2)  p=0.786  Time in Rescutation Room [min], mean (SD)  64 (±39)  72 (±40)  p<0.001  Time to admittance on ICU [min], mean (SD)  IG: 197 (±147)  CG: 197 (±149)  p=1.0  Time to arrival in the Operation Room [min], mean (SD)  IG: 141(±203)  CG: 144 (±187)  p=0.687  Length of Hospitalization [d], mean (SD)  IG: 27.9 (±30.2)  CG: 25.2 (±25.2)  p<0.001  Length of ICU stay [d], mean (SD)  IG: 13.0 (±15.4)  CG: 12.3 (±14.3)  p<0.01  RISC, mean (SD)  IG: 21.4 % (±28)  CG: 21.4 % (±28)  p=0.961  SMR  IG: 0.86  CG: 0.85  p=0.910 | Level of evidence  2b  Risk of bias  Selection bias: ?  Performance bias: ?  Attrition bias: +  Detection bias: +  Authors’ conclusion  „Emergency room protocols that use initial conventional radiographs prior to WB-MSCT have comparable results regarding mortality compared to protocols that use WBMSCT as the initial diagnostic tool. Furthermore, the emergency room team can perform life-savings procedures like chest-tube insertion, thoracotomy, and cardiopulmonary resuscitation immediately. Especially in patients in extremis, surgical procedures and diagnostic work-up can be performed simultaneous without wasting precious time.”  Reviewers’ conclusion  There is a risk of selection bias in this study and unclear performance bias. Besides that, there is high deviation within the reported outcomes (high standard deviations), results should be interpreted with caution. |
| +: low risk; –: high risk; ?: unclear risk; AIS: Abbreviated Injury Scale; CI: Confidence Interval; IQR: Interquartile Range; ISS: Injury Severity Score; OR: Odds Ratio; RR: Relative Risk; SD: Standard Deviation; PPV: Positive Predictive Value; NPV: Negative Predictive Value; AUC: Area under the curve; +LR: Positive Likelihood Ratio; -LR: Negative Likelihood Ratio; adj.: adjusted; d: days; m: months; y: years; min: minutes; RISC: Revised Injury Severity Calssification; SMR: Standardized Mortality Ratios; WB-MSCT: Whole Body-Multislice Computer Tomography | | | | |

##### Localization of the computed tomography (CT) scanner

| Study: Reference, aim, design, setting | Participants: selection criteria, characteristics | N Participants;  Intervention (IG) vs. Control group (CG) | Main outcomes | Assessment: LoE, risk of bias; Conclusions |
| --- | --- | --- | --- | --- |
| Wulffeld (2017)  “The effect of CT scanners in the trauma room - an observational study”. *Acta Anaesthesiologica Scandinavica* 2017; 61(7): 832-840  Study design  Retrospective before–after study  Aim of the study  „A CT scanner incorporated in the trauma resuscitation bay may benefit trauma patients by fastening work-up times; however, evidence in the area is still sparse. We assessed if time from admission to first CT scan was lower after incorporation of a CT scanner in the resuscitation bay.”  Setting  Denmark, 2011-2012 & 2013-2014 | Inclusion criteria   - Trauma patients in two 1-year periods before and after the reconstruction of the rescutation room, which took place from June 2012 to October 2013. - Patients who triggered trauma team activation.   Exclusion criteria   - Patients younger than 18 years - Burn patients   Characteristics  Age [y], median (IQR)  IG: 43 (29–59)  CG: 38 (26–55)  p=0.001  Male, n (%)  IG: 555 (74.8%)  CG: 561 (71.6)  p=0.2  ISS <15, n (%)  IG: 147 (19.8)  CG: 176 (22.5)  p=n.r.  ISS 16-24, n (%)  IG: 123 (16.6)  CG: 129 (16.5)  p=n.r.  ISS 25-49, n (%)  IG: 123 (16.6)  CG: 92 (11.7)  p=n.r.  ISS 50-75, n (%)  IG: 8 (1.1)  CG: 3 (0.4)  p=n.r.  First GCS<9, n (%)  IG: 134 (19.7)  CG: 99 (13.9)  TBI (AIS>3), n (%)  IG: 141 (19)  CG: 119 (15.2)  CT scans performed, n (%)  IG: 643 (86.7)  CG: 667 (85)  p=0.4  Urgent surgery, n (%)  IG: 148 (20)  CG: 160 (20.4)  p=0.8  Urgent surgery (subgroup ISS >15), n (%)  IG: 86 (33.9)  CG: 97 (43.3)  p=0.03 | Participants  N=1,526 trauma patients, 1,310 analyzed  Study groups  IG: after reconstruction, mobile CT scanners with a moving gantry (combined with a trauma resuscitation table) placed in rescutation room; N=742  CG: before reconstruction, CT scanner in a room 15m from the trauma room; N=784  Study interventions   - The before period went from 1 June 2011 to 31 May 2012 and the after period from 1 January 2014 to 31 December 2014. We chose two 1-year periods to eliminate bias due to seasonal variations. - We allowed a 3-month period between completion of the rebuilding and the after period to avoid bias due to start-up difficulties. | Primary outcomes^§^  30-day mortality, n (%)*  IG: 45 (6)  CG: 28 (3.6)  p=0.006  *Unadjusted  Adjusted OR, (95% CI)  1.1 (0.59-2.05)  Time to first CT image [min], median (IQR)  IG: 21 (17–28)  CG: 20 (15–29)  p=0.008,  HL median difference (95% CI)  1 (0-2)  Trauma room length of stay [min], median (IQR)  IG: 95 (67–136)  CG: 83 (60–129)  p<0.0001,  HL median difference, (95% CI):  10 (5–15)  Time to urgent surgery [min], median (IQR)  IG: 126.5 (78–209.5)  CG: 143 (92.5–230.5)  p=0.1,  HL median difference, (95% CI):  -18 (-40–3)  Subgroup patients with injury severity score >15^§^  Time to first CT image [min], median (IQR)  IG: 23 (18–32)  CG: 22 (16–34)  p=0.4,  HL median difference, (95% CI):  1 (-1–3)  Trauma room length of stay [min], median (IQR)  IG: 105 (78–141)  CG: 103 (67–146)  p=0.2,  HL median difference, (95% CI):  6 (-4–15)  Time to urgent surgery [min], median (IQR)  IG: 123 (85–183)  CG: 151 (100–224)  p=0.06,  HL median difference, (95% CI):  -24 (-49–2) | Level of evidence  2b  Risk of bias  Selection bias: +  Performance bias: ?  Attrition bias: +  Detection bias: +  Authors’ conclusion  „However, our results add to the current body of evidence where previous studies have found minor time gains only and no clear benefit in terms of patient outcomes. At this point, we think it is difficult to say if there is a clear benefit associated with having CT scanners in the trauma room.”  Reviewers’ conclusion  Results need to be interpreted with caution due to the retrospective before-after design of the study. Risk of performance bias is unclear. Results of the study showed no significant benefits which was already considered by the authors in their conclusion. |
| +: low risk; –: high risk; ?: unclear risk; CI: Confidence Interval; ISS. Injury Severity Score; HL: Hodges Lehmann estimate of the median difference; IQR: Interquartile Range; OR: Odds Ratio; RR: Relative Risk; SD: Standard Deviation; PPV: Positive Predictive Value; NPV: Negative Predictive Value; AUC: Area under the curve; +LR: Positive Likelihood Ratio; -LR: Negative Likelihood Ratio; adj.: adjusted; d: days; m: months; y: years; min: minutes | | | | |

##### Whole-body CT with contrast in hemodynamically unstable severely injured patients.

| Study: Reference, aim, design, setting | Participants: selection criteria, characteristics | N Participants;  Intervention (IG) vs. Control group (CG) | Main outcomes | Assessment: LoE, risk of bias; Conclusions |
| --- | --- | --- | --- | --- |
| Cook (2015)  “An Abdominal CT may be Safe in Selected Hypotensive Trauma Patients with Positive FAST Exam”. *American Journal of Surgery* 2015; 209(5): 834–840  Study design  Prospective cohort study  (database created by the Prospective Observational Multicenter Major Trauma Transfusion (PROMMTT) study Data Coordinating Center at the University of Texas Health Science Center at Houston)  Aim of the study  „We therefore sought to determine if patients who were initially hypotensive who undergo an abdomen and pelvis CT (CT) scan following a positive FAST exam have similar long term outcomes and less urgent operations than patients who do not undergo a CT.”  Setting  US, year n.r. | Inclusion criteria   - Patients who required the highest level activation at one of 10 Level I trauma centers & who received one or more units of red blood cells (RBCs) within 6 hours of hospital admission (PROMMTT inclusion criteria) - Patients with a positive FAST and hypotension defined as an admission systolic blood pressure (SBP) ≤90mmHg (studies inclusion criteria)   Exclusion criteria   - age younger than 16 years - transfer from another hospital - pregnancy - more than 20% burn injury - inhalation injury - incarceration - cardiopulmonary resuscitation lasting more than 5 minutes occurring prehospital or in the first 30 minutes after admission - death within 30 minutes of hospital admission   Characteristics  Age [y], median (IQR)  IG: 34 (24 - 45)  CG: 41 (24 - 54), p=0.15  Male, n (%)  n.r.  GCS, median (IQR)  CG: 9 (4 - 14)  IG: 13 (3 - 5), p=0.15  ISS, median (IQR)  CG: 34 (23 - 41)  IG: 27 (16 - 35), p=0.12  Head AIS, median (IQR)  IG: 0.5 (0 - 4)  CG: 0 (0 - 0.8), p<0.05  Face AIS, median (IQR)  IG: 0 (0 - 1)  CG: 0 (0 - 0), p=0.05  Chest AIS, median (IQR)  IG: 3 (3 - 4)  CG: 3 (1 - 4), p=0.19  Abdomen AIS, median (IQR)  IG: 3 (3 - 4)  CG: 3 (2 - 4), p=0.8  Extremity AIS, median (IQR)  IG: 2 (0 - 3)  CG: 2 (0 - 3), p=0.82  External AIS, median (IQR)  IG: 1 (0 - 1)  CG: 1 (0 - 1), p=0.62 | Participants  N=255 patients, 92 analyzed  Study groups  IG: Patients with positive FAST, hypotension and CT diagnostic; N=32  CG: Patients with positive FAST, hypotension and without CT diagnostic; N=60  Study interventions   - CT was defined as Abdominal/Pelvis CT scan - An urgent operation was defined as direct admission to the operating room less than 3 hours from presentation. This longer time was chosen to allow for the inherent delay in obtaining a CT scan during trauma workup.   Criteria for adjustment, multivariable model   - Age - GCS - ISS - Admission systolic blood pressure - Heart rate - Mechanism of injury | Primary outcomes  Urgent Operation, %; OR (95% CI)  IG: 22  CG: 93; 0.02 (95% CI: <0.01 – 0.15)*; p<0.01  *CT was associated with reduced odds of an urgent operation  Urgent Angiography, %; OR (95% CI)  IG: 22  CG: 2; 15.8 (1.5–133.2)*; p<0.01  *CT was associated with increased odds of proceeding to interventional radiography.  Time to Operation [min], median (95% CI)  IG: 93 (41 - 121)  CG: 26 (19 - 35), p<0.01  24-h mortality, % OR (95% CI)  IG: 6  CG: 20, p<0.01  0.41 (0.05–3.6)  30 Day Mortality, % OR (95% CI)  IG: 19  CG: 30, p=0.32  1.4 (0.24–7.7)  Length of stay, median (IQR)  IG: 20 (14 - 37)  CG: 17 (11 - 30), p=0.49 | Level of evidence  2b  Risk of bias  Selection bias: ?  Performance bias: ?  Attrition bias: +  Detection bias: +  Authors’ conclusion  “In conclusion, we find that some patients with initial hypotension and a positive FAST may be taken to CT without a significant difference in 30 day mortality. We additionally find that undergoing an CT is independently associated with reduced odds of an urgent operation and increased odds of angiographic intervention. This suggests that the information obtained from the CT may impact clinical decisions and that admission hypotension and a positive FAST exam does not mandate laparotomy.”  Reviewers’ conclusion  There is an unclear risk of selection and performance bias as the study groups remained different concerning the ISS although attempts to reduce confounding factors were made.  Furthermore, the patient selection process remains unclear as more patient data were selected for the PROMMTT database but only a minority was analyzed. Reason for the selection of the other patients was not explained. |
| Katayama (2018)  “Delay of computed tomography is associated with poor outcome in patients with blunt traumatic aortic injury (BTAI) - A nationwide observational study in Japan” *Medicine*, 2018. 97(35): p. e12112.  Study design  Comparative registry study  (Japanese Trauma Data Bank)  Aim of the study  “The aim of this study was to assess the relationship between the timing of CT scanning and the prognosis of BTAI patients using this database.”  Setting  Japan, 2004-2015 | Inclusion criteria   - Emergency patients who had a BTAI in the chest and/or the abdomen among those who were transported to the JTDB-participating hospitals and were registered in the database.   Exclusion criteria   - patients with cardiopulmonary arrest on hospital arrival - patients with interhospital transport - patient which had no records on the time interval from hospital arrival to CT scanning - patients with penetrating trauma - inappropriate datasets - patients in whom the first elective CT scanning was performed ≥72hours after hospital arrival   Characteristics  Age[y], median (IQR)  IG1: 59 (38–71)  IG2: 57 (37–73)  CG: 62 (47–77), p=0.496  Male, n (%)  IG1: 93 (68.9)  IG2: 106 (73.6)  CG: 109 (76.8), p=0.332  ISS, median (IQR)  IG1: 34 (24–48  IG2: 35 (26–50)  CG: 34 (25–50), p=0.451  Shock (systolic blood pressure<90mmHg), n (%)  IG1: 48 (35.6)  IG2: 52 (36.1)  CG: 37 (26.1), p=0.128  Surgical operation, n (%)  IG1: 20 (14.8)  IG2: 34 (23.6)  CG: 28 (19.7), p=0.179  Time interval from patient’s call to hospital arrival [min], median (IQR)  IG1: 38 (29–47)  IG2: 38 (29–51)  CG: 38 (28–54), p=0.405 | Participants  N=421 BTAI patients  Study groups  IG1: Time interval from hospital arrival to CT scanning; 27-40 min; N=135  IG2: Time interval from hospital arrival to CT scanning; >=41 min; N=144  CG: Time interval from hospital arrival to CT scanning; =<26 min; N=142  Study interventions  The tertile groups by CT scanning time were classified according to the time interval from hospital arrival to implementation of first CT scanning by a CT operator. | Primary outcomes  Death in the ED, n (%)  IG1: 15 (11.1); adj. OR*= 1.833, 95% CI: 0.601 – 5.590; p=0.287  IG2: 25 (17.6); adj. OR*= 2.832, 95% CI: 1.007 – 7.960; p=0.048  CG: 11 (7.7)  * Adjusted for: age, gender, falling from a high place, pedestrian injured by traffic accident,  calendar year, time of the day (daytime/nighttime), day of the week (weekday/weekend and holiday), shock at hospital arrival, RTS, ISS, case volume (upper/middle/lower)  Death to discharge, n (%)^§^  IG1: 43 (31.9); adj. OR^1^= 1.032 , 95% CI: 0.517 – 2.059; p=0.930  IG2: 55 (38.2); adj. OR^1^= 1.438 , 95% CI: 0.735 – 2.813; p=0.288  CG: 41 (28.9)  ^1^Adjusted for age, gender, time of the day, day of the week, falling from a high place, pedestrian injured by traffic accident, revised trauma score, injury severity score, shock at hospital arrival, case volume, and calendar year.  Subgroup: Death in the ED of BTAI patients with and without shock according to CT scanning time^§^  *Shock (n=137)*  Death in the ED, n (%)  IG1: 10 (20.8); adj. OR^2^= 3.292; 95% CI: 0.495 – 21.902; p=0.218  IG2: 14 (26.9); adj. OR^2^= 6.039; 95% CI: 0.990 – 36.837; p=0.051  CG: 5 (13.5)  *No shock (n=284)*  Death in the ED, n (%)  IG1: 5 (5.7); adj. OR^2^= 1.527; 95% CI: 0.337 – 7.103; p=0.575  IG2: 11 (12); adj. OR^2^= 2.165; 95% CI: 0.533 – 8.785; p=0.280  CG: 6 (5.7)  ^2^Adjusted for age, gender, time of the day, day of the week, falling from a high place, pedestrian injured by traffic accident, revised trauma score, injury severity score, case volume, and calendar year.  ^§^findings without significance | Level of evidence  2b  Risk of bias  Selection bias: +  Performance bias: ?  Attrition bias: +  Detection bias: +  Authors’ conclusion  “We showed in a retrospective review of a nationwide hospital based trauma registry in Japan that the prognosis of BTAI patients in the ED worsened as the time to first CT scanning was delayed.”  Reviewers’ conclusion  Results need to be interpreted with caution due to the retrospective design of the study and the unclear risk of performance bias. There is only one significant finding: there is an increase of deaths in the ED upon patients receiving CT scanning later compared to early scan. The other findings were not significant. Furthermore, results show wide 95% confident intervals. |
| Tsutsumi (2017)  „Computed tomography during initial management and mortality among hemodynamically unstable blunt trauma patients: a nationwide retrospective cohort study”. *Scandinavian Journal of Trauma, Resuscitation and Emergency Medicine* 2017; 25:74  Study design  Comparative registry study  (Japan Trauma Data Bank)  Aim of the study  „In this study, we examined the association between CT and mortality among unstable blunt trauma patients using nationwide Japanese registry data, to clarify whether CT has harmful effect among these patients after adjusting both for measured and unmeasured confounders.”  Setting  Japan, 2004–2014 | Inclusion criteria   - all blunt trauma patients with hypotension (SBP <90 mmHg) on arrival at the emergency department (ED) during the study period   Exclusion criteria   - aged <16 years - patients who were transferred from other facilities - patients who suffered cardiac arrest (no heart rate; no respiratory rate; and no palpable pulse) or near-arrest (SBP ≤40 mmHg; based on the JTDB registration criteria that blood pressure cannot be measured at 40 mmHg SBP but a pulse is palpable) on arrival. - patients with missing data on items such as prognosis, CT information, and date of admission - patients with missing data of any covariate necessary for the multivariate analysis - patients treated in facilities that had small volumes of eligible patients (<10 patients)   Characteristics  Age [y], mean (±SD)  IG: 56.2 (21.8)  CG: 58.7 (20.6)  p=0.013  Male, n (%)  IG: 3,535 (66.1)  CG: 278 (60.8)  p=0.024  Systolic Blood pressure on arrival [mmHg], mean (SD)  IG: 75.8 (10.0)  CG: 73.6 (11.1)  p<0.001  Heart Rate [per min], mean (SD)  IG: 92.9 (27.7)  CG: 99.2 (31.2)  p<0.001  GCS, mean (SD)  IG: 11.4 (4.3)  CG: 10.9 (4.8)  p=0.008  ISS, mean (±SD)  IG: 26.2 (15.1)  CG: 23.9 (16.8)  p=0.002 | Participants  N=5,809 data sets of blunt trauma patients  Study groups  IG: CT group, defined as CT of at least one body region; whole-body CT and those undergoing selective CT were all included; N=5,352  CG: no CT received; N=457 | Primary outcomes  In-hospital death, n (%)  IG: 1,276 (23.8)  CG: 207 (45.3)  p<0.001  Death within 24h, n (%)  IG: 655 (12.8)  CG: 147 (34.9), p<0.001  Number of excess deaths (per 100 patients) in-hospital mortality, n (95% CI)^§^  inverse probability of treatment weighted analysis: -20.6 (-26.2 to-14.9)  instrumental variable analysis: -4.1 (-23.1 to 14.8)  Number of excess deaths (per 100 patients) 24h-mortality, n (95% CI)^§^  inverse probability of treatment weighted analysis: −20.9 (−26.4 to −15.5)  instrumental variable analysis: −13.6 (−30.6 to 3.4)  ^§^ age, gender, systolic blood pressure, heart rate and Glasgow Coma Scale, Injury Severity Score and year of injury were included as covariate in both analyses | Level of evidence  2b  Risk of bias  Selection bias: +  Performance bias: ?  Attrition bias: +  Detection bias: +  Authors’ conclusion  „In summary, most unstable blunt trauma patients undergo CT as part of initial management in Japan. We did not find clinically meaningful harmful effect of CT on survival for unstable blunt trauma patients after adjusting both for measured and unmeasured confounders. Our results do not support the current guidelines, of which only a few recommend CT for unstable patients.”  Reviewers’ conclusion  Results need to be interpreted with caution due to the retrospective design of the study. The authors integrated covariates and confounding factors in their analysis. The sampler sample size of the control (no CT group) was adjusted by calculating a pseudo-population of no-CT group with the same size as the intervention group. |
| +: low risk; –: high risk; ?: unclear risk; CI: Confidence Interval; IQR: Interquartile Range; OR: Odds Ratio; SD: Standard Deviation; PPV: Positive Predictive Value; NPV: Negative Predictive Value; AUC: Area under the curve; +LR: Positive Likelihood Ratio; -LR: Negative Likelihood Ratio; adj.: adjusted; d: days; m: months; y: years; min: minutes | | | | |

##### Prehospital sonography

| Study: Reference, aim, design, setting | Participants: selection criteria, characteristics | N Participants;  Intervention (IG) vs. Control group (CG) | Main outcomes | Assessment: LoE, risk of bias; Conclusions |
| --- | --- | --- | --- | --- |
| Press (2014)  "Prospective Evaluation of Prehospital Trauma Ultrasound During Aeromedical Transport". *The Journal of Emergency Medicine* 2014*,* Vol. 47, No. 6, pp. 638–645.  Study design  Diagnostic cross-sectional study  Aim of the study  “The goal was to assess prehospital provider accuracy in performing the abdominal, cardiac, and lung components of EFAST.”  Setting  USA, 7-month-study | Inclusion criteria   - adult trauma patients transferred directly from scene if time allowed after standard stabilization - age ≥18 years   Exclusion criteria  n.r.  Characteristics  Age [y], mean ± SD  41 ± 17  Male, n (%)  216 (74)  ISS mean ± SD  16 ± 11  Trauma type, n (%)  Blunt 252 (88.4)  Penetrating 33 (11.6)  Weight (kg), mean ± SD  82 ± 18  Scene systolic blood pressure (mm Hg), mean ± SD  130 ± 27  Scene heart rate (bpm), mean ± SD  94 ± 22  Base deficit, mean ± SD  3.1 ± 4.5  Transport time to ED (min), mean ± SD  20.9 ± 8.7  ED GCS  12 + 3/–4 | Participants  Adult trauma patients from scene N=833  Patients with at least one HEMS ultrasound n=293  Number of lung HEMS ultrasound n=511  Tests evaluated  Index text: In-flight ultrasound. HEMS providers were trained to perform EFAST during a 2-month period. HEMS providers performed EFAST using the following views: hepatorenal, splenorenal, suprapubic, cardiac (subcostal or parasternal long-axis), right lung, and left lung. All views were standard and in accordance with imaging described by the American College of Emergency Physicians and American Institute of Ultrasound in Medicine (19). Abdominal and cardiac examinations were performed to evaluate for intraperitoneal and pericardial fluid, respectively. Lung ultrasound was performed to evaluate for lung slide to exclude or diagnose pneumothorax. Abdominal views were saved as still images, and cardiac and lung views as 4-s video clips.  Reference standard: ED diagnostics and management including CT, chest radiography and clinical examination. | Primary outcomes  Diagnostic accuracy for detection of pneumothorax  true positive, n=8  false positive, n=2  true negative, n=444  false negative, n=35  Sensitivity, % (95% CI)  18.7 (8.9–33.9)  Specificity, % (95% CI)  99.5 (98.2–99.9),  PPV, % (95% CI)  80 (44.2–96.5),  NPV, % (95% CI)  92.7 (89.9–94.8)  Diagnostic accuracy for detection of pneumothorax that required intervention  true positive, n=9  false positive, N=1  true negative, n=469  false negative, n=0  Sensitivity, % (95% CI)  50 (22.3–58.7)  Specificity, % (95% CI) , n/N  99.8 (98.6–100), 469/470  PPV, % (95% CI)  90 (54.1–99.5)  NPV, % (95% CI)  98.1 (96.3–99.1) | Level of evidence  2b  Risk of bias (QUADAS)  Patient selection: +  Index test: +  Reference standard: +  Flow and timing: +  Authors’ conclusion  “Positive interpretations significantly raised the probability of injury, more reliably so for lung ultrasound. Negative interpretations were predictive, but low prevalence limited the value of these results. Sensitivity was not sufficient for ruling out injury. We believe further study is needed to elucidate accuracy as providers gain experience, and to explore clinical outcomes that may be affected by prehospital trauma ultrasound.”  Reviewers’ conclusion  HEMS providers were new to inflight ultrasound and received a training. The guidance from this training may have a high influence on current behaviour. Also all staff knew about the study which may have introduced a Hawthorne effect. |
| Quick (2016)  "In-flight ultrasound identification of pneumothorax". *Emerg Radiol* (2016) 23:3–7.  Study design  Diagnostic cross-sectional study  Aim of the study  “Our study sought to demonstrate the accurate and timely detection of correctable thoracic pathology, specifically pneumothorax and improperly positioned endotracheal tubes by non-physician, prehospital flight crews trained in the use of thoracic ultrasound.”  Setting  USA, 15-month | Inclusion criteria   - all adult trauma patients, - all intubated adult medical patients transported by one of University of Missouri’s Staff for Life Helicopters   Exclusion criteria  n.r.  Characteristics  Age [y], mean (range)  44.4 (16-94)  Male, n (%)  133 (69)  ISS mean (range)  17.68 (1-75)  Chest AIS mean (range)  2.93 (0-6)  BMI mean (range)  28.2 (15–50) | Participants  N=149 patients receiving in-flight ultrasound  N=116 patients receiving CT scan  Tests evaluated  Index text: In-flight ultrasound. Twenty-six flight crew members were trained to perform and interpret thoracic ultrasound prior to the initiation of the study. Flight crews recorded their interpretations of radiographic findings using an evaluation form.  Reference standard: CT scan. Routine clinical care was provided in accordance with ATLS methods to include the completion of an E-FAST by the trauma team. Further imaging was obtained as needed during patient evaluation. Computed tomography (CT) was considered the criterion standard and utilized to confirm either the presence or absence of pneumothorax and proper endotracheal tube placement. Patients that did not undergo CT evaluation had either clearly visible pneumothorax on chest X-ray or definitive clinical signs of a pneumothorax. | Primary outcomes  Diagnostic test performance for detecting pneumothorax  true positive, n=16  false positive, n=1  true negative, n=129  false negative, n=3  Accuracy, % (95% CI)  91 (0.85-0.95)  Sensitivity, % (95% CI)  68 (0.46–0.85)  Specificity, % (95% CI)  96% (CI 0.90–0.98)  PPV, % (95% CI)*  94.1  *calculated with true positive/ all positives  NPV, % (95% CI)*  97.7  *calculated with true negatives/ all negatives | Level of evidence  2b  Risk of bias (QUADAS)  Patient selection: +  Index test: +  Reference standard: +  Flow and timing: ?  Authors’ conclusion  “Ultrasonography should be utilized to augment the diagnostic capabilities of all prehospital aeromedical providers. Routine use of in-flight ultrasound is one step closer to getting the right care to the right patient at the earliest possible instance and could lead to better outcomes. A multicenter trial is warranted to further confirm this benefit.”  Reviewers’ conclusion  Providers were new to inflight ultrasound and received a training. The guidance from this training may have a high influence on current behaviour. It is unclear how many patients received only chest radiography as reference standard. |
| +: low risk; –: high risk; ?: unclear risk; CI: Confidence Interval; IQR: Interquartile Range; OR: Odds Ratio; RR: Relative Risk; SD: Standard Deviation; SEM: Standard Error of Mean; PPV: Positive Predictive Value; NPV: Negative Predictive Value; AUC: Area under the curve; +LR: Positive Likelihood Ratio; -LR: Negative Likelihood Ratio; adj.: adjusted; d: days; m: months; y: years; min: minutes | | | | |

##### Whole body CT

| Study: Reference, aim, design, setting | Participants: selection criteria, characteristics | N Participants;  Intervention (IG) vs. Control group (CG) | Main outcomes | Assessment: LoE, risk of bias; Conclusions |
| --- | --- | --- | --- | --- |
| Treskes (2020)  “Refining the criteria for immediate total-body CT after severe trauma.” *European Radiology*, 2020. 30(5): p. 2955-2963.  Study design  Secondary analysis of an RCT (Sierink 2016)  (five trauma centers)  Aim of the study  „The aim of the present analysis was to assess the discriminatory power of REACT-2 criteria for severely injured patients that could benefit from immediate total-body CT (iTBCT) during the primary assessment of trauma care. Furthermore, a revised set of criteria was derived and tested for discriminatory characteristics on detection of severe injury and shifts in radiation exposure compared to the original set of REACT-2 inclusion criteria.”  Setting  The Netherlands and Switzerland, 2011 -2014 | Inclusion criteria   - Trauma patients with the presence of life-threatening vital problems, at least one of the following: respiratory rate ≥30 min or≤10/min; pulse ≥120/min; systolic blood pressure ≤100 mmHg; estimated exterior blood loss ≥500 ml; Glasgow Coma Score ≤13; Abnormal pupillary reaction onsite. - patients with one of the following clinically suspicious diagnoses: flail chest, open chest or multiple rib fractures; severe abdominal injury; pelvic fracture; unstable vertebral fractures/spinal cord compression; fractures from at least two long bones - patients with one of the following injury mechanisms: fall from height (>3 m/>10 ft); ejection from the vehicle; death occupant in same vehicle; severely injured patient in same vehicle; wedged or trapped chest/abdomen.   Exclusion criteria   - known age <18 years - known pregnancy - referred from another hospital - clearly low-energy trauma with blunt injury mechanism - penetrating injury in 1 body region (except gunshot wounds) as the clearly isolated injury - any patient who is judged to be too unstable to undergo a CT scan and requires (cardiopulmonary) resuscitation or immediate operation because death is imminent according to the trauma team leader in mutual agreement with the other leading care givers.   Characteristics  Age [y], median (IQR)  43 (27–59)  Male sex, n (%)  824 (76.1)  In hospital GCS, median (IQR):  13 (3–15)  Abbreviated Injury Scale ≥3, n (%)  Head: 465 (42.9)  Chest: 435 (40.2)  Abdomen: 116 (10.7)  Arms, legs, hand, and feet: 304 (28.1)  ISS, median (IQR):  20 (9–29)  Patients with polytrauma, n (%)  693 (64.0)  Patients with traumatic brain injury, mean (SD):  329 (30.4) | Participants  N=1,083 patients  Study groups/ original REACT-2 iTBCT criteria  Respiratory rate ≥30/min or ≤10/min: n=16  Pulse ≥120/min: n=69  Pulse ≥130/min†: n=49  Pulse ≥140/min†: n=26  Systolic blood pressure ≤100 mmHg: N=116  Systolic blood pressure <90 mmHg†: n=82  Systolic blood pressure <80 mmHg†: n=32  Estimated exterior blood loss ≥500 ml: n=43  GCS ≤13 or abnormal pupillary reaction: n=485  GCS ≤8†: n=437  GCS = 3†: n=394  Fractures from at least two long bones: n=90  Flail chest, open chest, or multiple rib fractures: n=114  Severe abdominal injury: n=65  Pelvic fracture: n=98  Unstable vertebral fractures/spinal cord compression: n=69  Fall from height (>3 m/>10 ft): n=319  Fall from height (>4 m/>13 ft)†: n=166  Fall from height (>5 m/>16 ft)†: n=126  Fall from height (>6 m/>20 ft)†: n=82  Fall from height (>7 m/>23 ft)†: n=60  Fall from height (>8 m/>26 ft)†: n=40  Ejection from a vehicle: n=30  Death of occupant in same vehicle: n=17  Severely injured patient in same vehicle: n=18  Wedged or trapped chest/abdomen: n=60  Note  By logistic regression analysis with backward selection on the 15 study inclusion criteria, a revised set of criteria was derived and subsequently tested for prediction of severe injury and shifts in radiation exposure.  When clinically appropriate, the threshold values for vital parameters and trauma mechanism characteristics of specific criteria were retrospectively adjusted and included again in the regression analysis. | Primary outcomes  Selected adjusted REACT-2 iTBCT criteria  N=10  Prognostic performance of the adjusted criteria (overall performance)  Number needed to overscan†, n (95% CI)  5.6 (4.9–6.5)  Decrease of unnecessary iTBCT-scans‡, % (95% CI):  6 (2–10)  PPV, % (95% CI):  82 (80–85)  Relative sensitivity*, % (95% CI):  91% (89–93)  AUC ROC, (95% CI):  0.80 (0.77–0.83)  Performance of each selected criteria  Selected adjusted criteria, OR (95% CI):  Systolic blood pressure ≤100 mmHg (n=116) 5.72 (2.22–14.75), p<0.001  Estimated exterior blood loss ≥500 ml (n=43) 3.70 (1.20–11.37), p=0.023  GCS ≤13 or abnormal pupillary reaction (n=485) 12.65 (8.23–19.45), p<0.001  Fractures from at least two long bones (n=90) 4.94 (2.41–10.15), p<0.001  Flail chest, open chest, or multiple rib fractures (n=114) 3.27 (1.85–5.76), p<0.001  Pelvic fracture (n=98) 1.82 (1.05–3.14), p=0.033  Unstable vertebral fractures/spinal cord compression (n=69) 1.87 (1.06–3.31), p=0.032  Fall from height (>4 m/>13 ft)* (n=166) 1.64 (1.07–2.52), p=0.022  Wedged or trapped chest/abdomen (n=60) 2.57 (1.20–5.51), p=0.015  †Number of iTBCT scans to perform one unnecessary iTBCT for a non-severely injured patient  ‡ Percentage decrease of iTBCT scans for non-severely injured patients  *Relative sensitivity within the population preselected by the original criteria | Level of evidence  2b  Risk of bias  no tool available for prognostic studies  Authors’ conclusion  “This study presents a revised set of 10 clinically criteria for iTBCT with a high predictive value for severe injury and therefore reduces radiation for the less severely injured patients for iTBCT. The criteria selected as predictors in this study should be prospectively validated in another cohort of patients for whom screening by iTBCT is considered after severe trauma.”  Reviewers’ conclusion  The study was a secondary analysis on prognostic factors of a well conducted RCT. There was a lack of information on patients who were not selected by the original REACT-2 criteria for eligibility of screening by iTBCT. This study could therefore only report the relative reduction of the sensitivity by the revised set compared to the original set of criteria. |
| +: low risk; –: high risk; ?: unclear risk; CI: Confidence Interval;; IQR: Interquartile Range; OR: Odds Ratio; RR: Relative Risk; SD: Standard Deviation; SEM: Standard Error of Mean; PPV: Positive Predictive Value; NPV: Negative Predictive Value; AUC: Area under the curve; +LR: Positive Likelihood Ratio; -LR: Negative Likelihood Ratio; adj.: adjusted; d: days; m: months; y: years; min: minutes | | | | |

# S5 Deleted Recommendations

| **2.5** | **Bildgebung** |  |  |  |
| --- | --- | --- | --- | --- |
| 2.129  0 | Bei hämodynamisch instabilen Schwerverletzten kann unter bestimmten Voraussetzungen* unmittelbar ein Ganzkörper-CT^1^ mit Kontrastmittel durchgeführt werden.  ^1^(Kopf bis einschließlich Becken, CCT nativ)  *Voraussetzungen hierfür sind ein hoher Organisationsgrad des Traumateams und die entsprechende Infrastruktur.  *Neu 2016* | 2.129 |  | Streichen den Empfehlung |
